# Supplementary material for: A unified model-based framework for doublet or multiplet detection in single-cell multiomics data
Source: Nat Commun. 2024 Jul 2;15:5562. doi: 10.1038/s41467-024-49448-x (PMC11220103; doi:10.1038/s41467-024-49448-x)
Supplement: Supplementary file 1 — Supplementary information file [file 41467_2024_49448_MOESM1_ESM.pdf]

Supplementary Figures

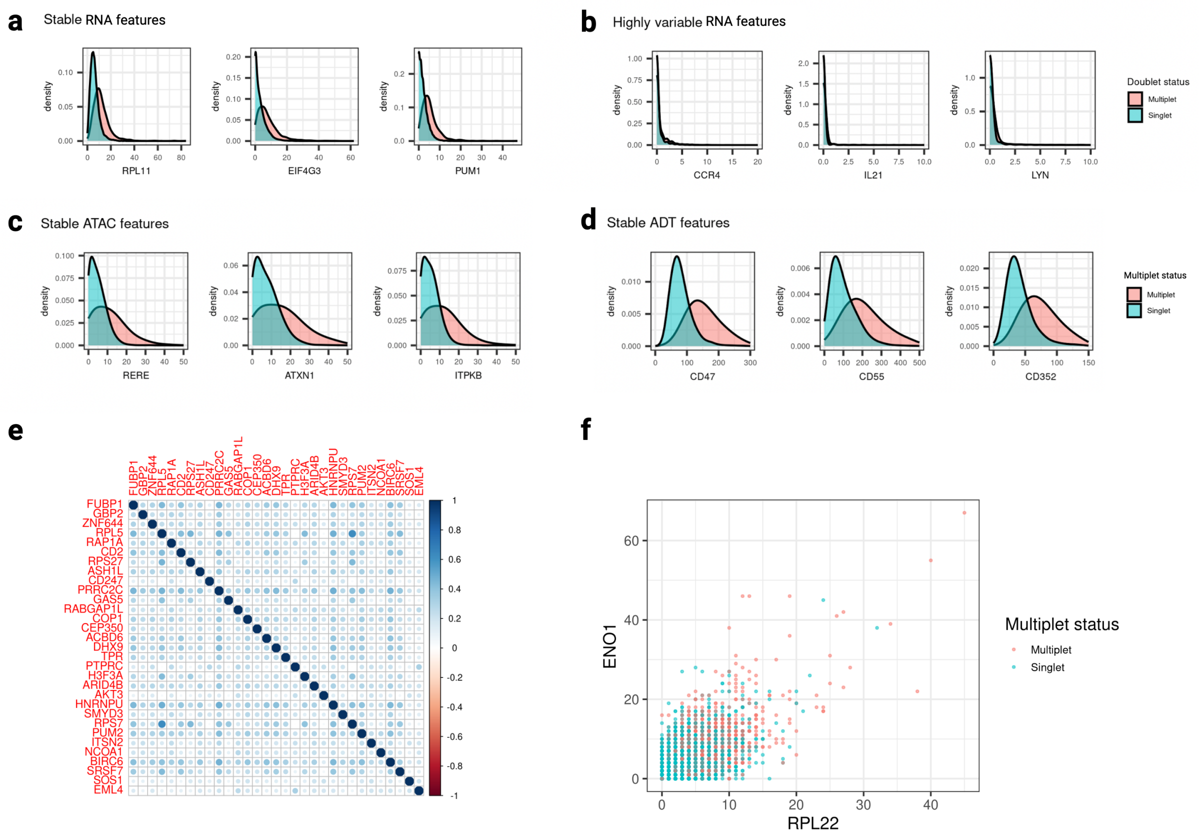

Figure S1: Illustrations of stable feature distributions and correlations

The data are from the PB-1 dataset.

a-b Density plots comparing the distributions of stable RNA features vs. highly variable RNA features stratified by multiplet status. The distributions of stable RNA features exhibit more obvious differences between singlets and multiplets. Although the single-cell data are discrete, the distributions of stable features are close to continuous distributions.

c-d Distributions of stable ATAC features (**c**) and stable ADT features (**d**) for singlets and multiplets.

e Correlation plot of stable genes within singlets. The correlations are close to zero.

f Scatter plot illustrating the correlations between two stable genes. Stratified on multiplet status, the distribution of the dots, which represent droplets, are close to random and no obvious correlation is observed. Note that some ground truth singlets in the upper-right part of the plot may be multiplets not identified by cell hashing. Source data are provided as a Source Data file.

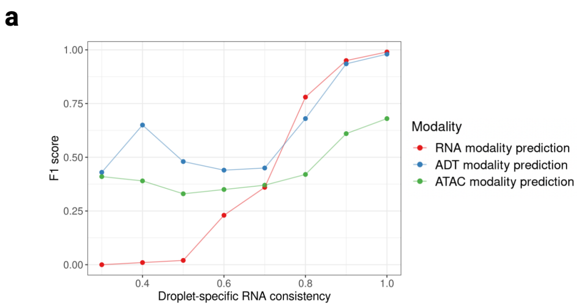

Figure S2: Relationship between modality consistency and single-modality COMPOSITE prediction performance

Prediction performance (in terms of F1 score) of each modality at different RNA (**a**) and ATAC (**b**) consistency levels. The data are from the Ileum-1 dataset. Source data are provided as a Source Data file.

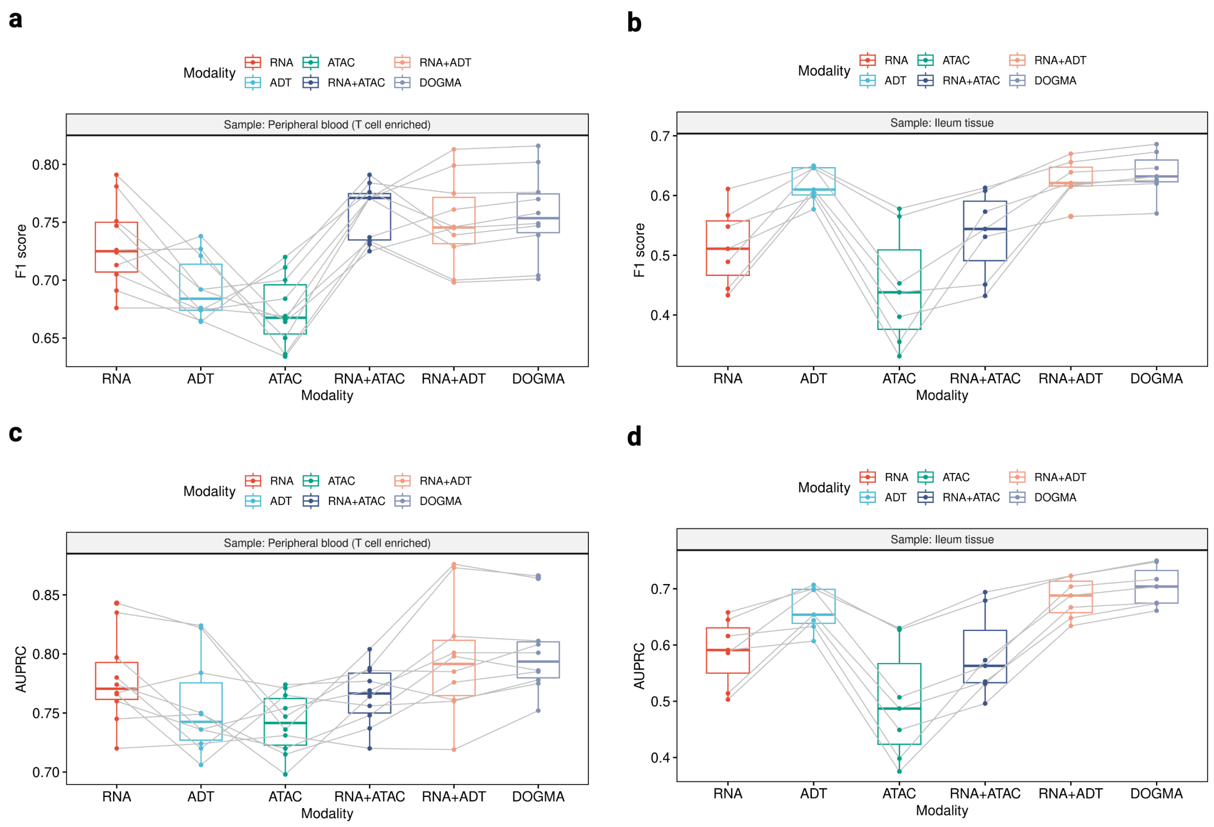

Figure S3: Comparing the F1 score and area under the precision-recall curve (AUPRC) of single-omics and multiomics COMPOSITE predictions

a-b Paired boxplots showing F1 scores achieved by COMPOSITE using different combinations of modalities from the in-house peripheral blood samples **(a)** and ileum biopsy samples **(b)**.

c-d Paired boxplots showing AUPRC values achieved by COMPOSITE using different combinations of modalities from the in-house peripheral blood samples (**c**) and ileum biopsy samples (**d**).

In the boxplots, the box spans from the first to third quartile, with the median depicted as a line in the middle. The whiskers extend to 1.5 times the interquartile range (IQR). Dots connected by grey lines represent the prediction achieved using different combinations of modalities from the same dataset. Source data are provided as a Source Data file.

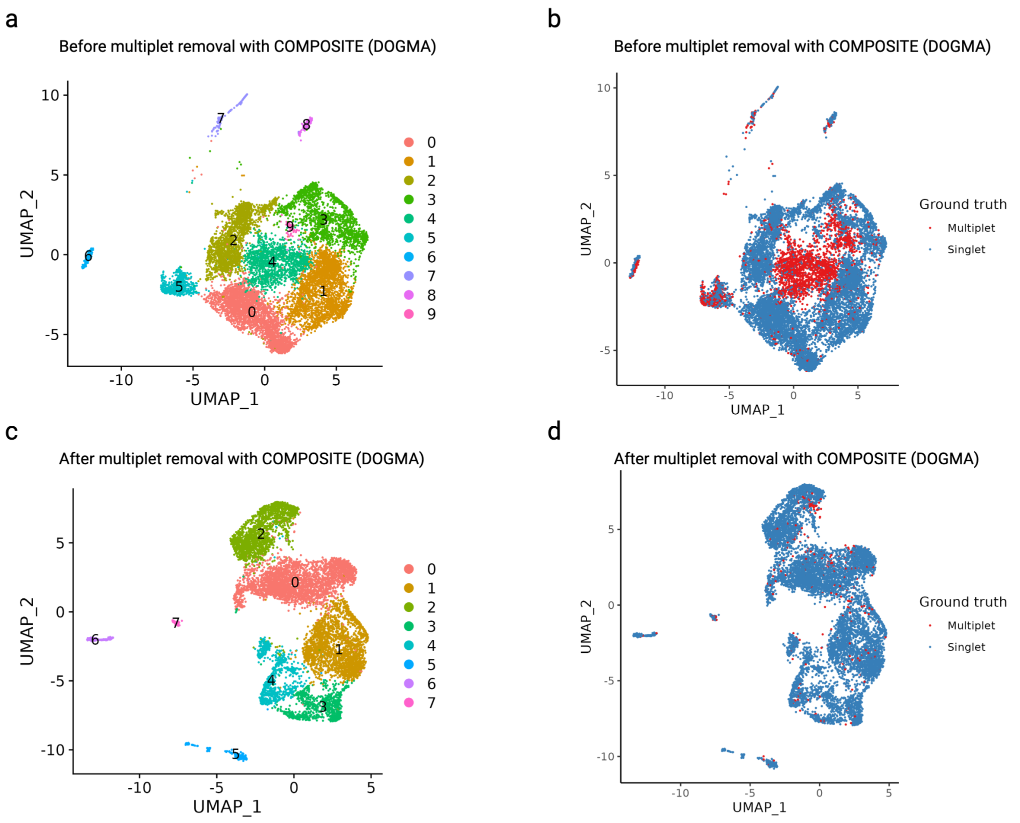

Figure S4: The impact of COMPOSITE (DOGMA) multiplet removal on the PB-1 dataset clustering results

a UMAP plot showing the clustering results before multiplet removal

b UMAP plot showing the ground truth multiplet status of each droplet before multiplet removal

c UMAP plot showing the clustering results after multiplet removal with COMPOSITE (DOGMA)

d UMAP plot showing the ground truth multiplet status of each droplet after multiplet removal with COMPOSITE (DOGMA). Source data are provided as a Source Data file.

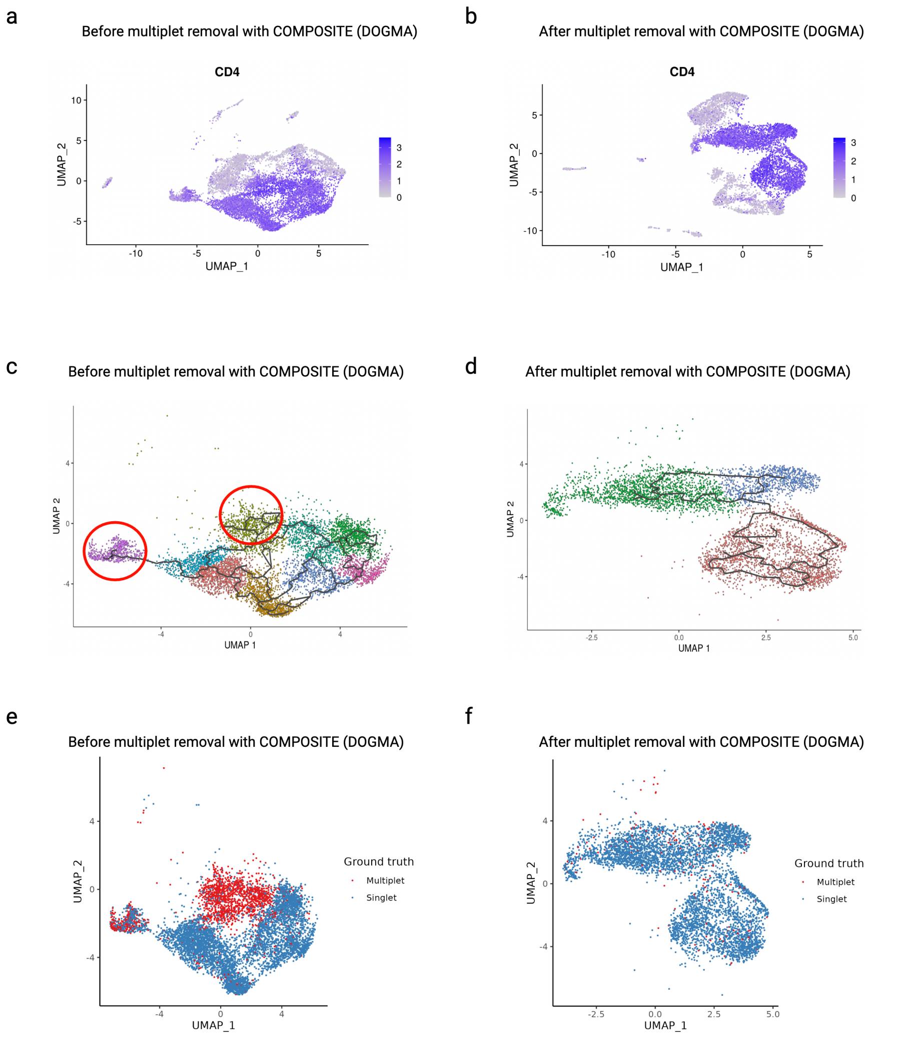

Figure S5: The Impact of Multiplet Removal by COMPOSITE (DOGMA) on Trajectory Inference for CD4+ T Cells in the PB-1 Dataset

a UMAP plot showing the CD4 surface protein marker expression of each droplet before multiplet removal.

b UMAP plot showing the CD4 surface protein marker expression of each droplet after multiplet removal with COMPOSITE (DOGMA).

c UMAP plot showing the trajectory inference on annotated CD4+ T cells before multiplet removal. The red circles mark the branches of the trajectory that extend into the multiplet clusters.

d UMAP plot showing the trajectory inference on annotated CD4+ T cells after multiplet removal with COMPOSITE (DOGMA).

e UMAP plot showing the ground truth multiplet status of the droplets that are annotated to be CD4+ T cells before multiplet removal.

f UMAP plot showing the ground truth multiplet status of the droplets that are annotated to be CD4+ T cells after multiplet removal with COMPOSITE (DOGMA). Source data are provided as a Source Data file.

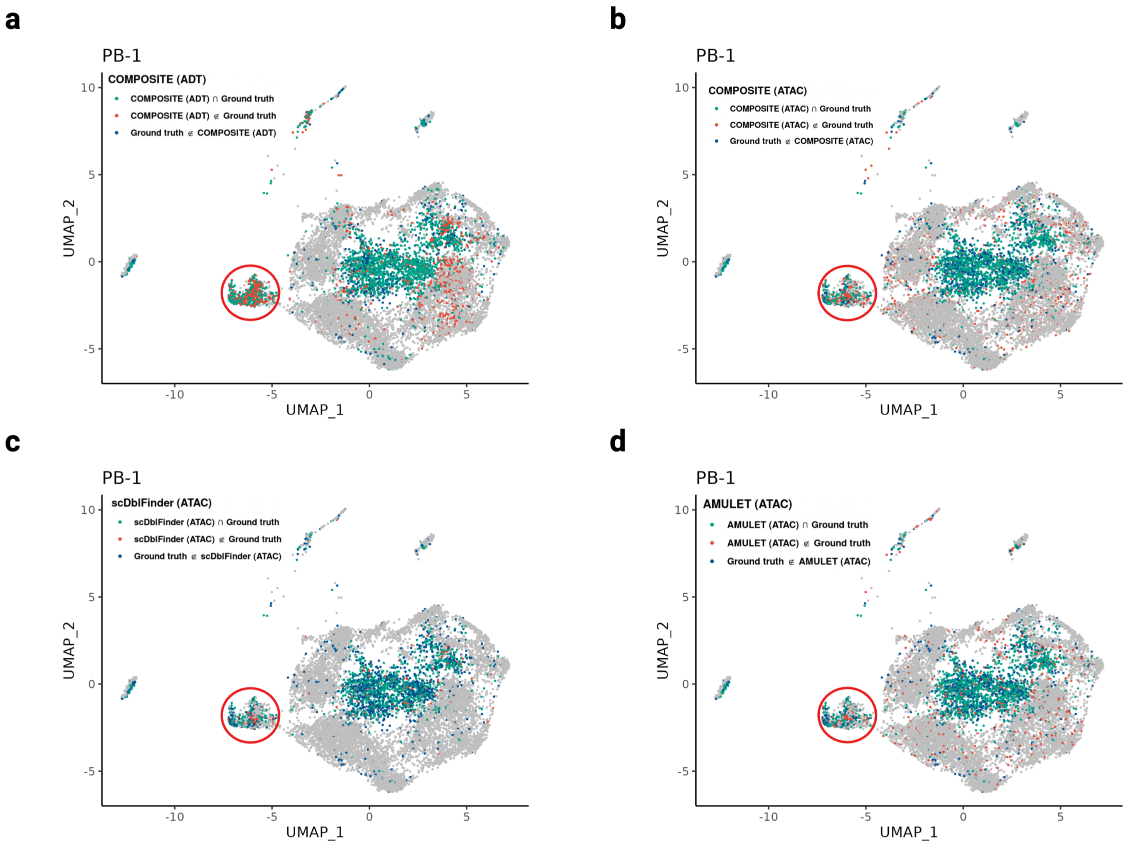

Figure S6: Supplementary benchmarking of multiplet prediction methods on the PB-1 dataset
a-d UMAP plots displaying the comparison between multiplet predictions and ground truth on the PB-1 dataset. The methods shown are COMPOSITE (ADT) (**a**), COMPOSITE (ATAC) (**b**), scDblFinder (ATAC) (**c**), and AMULET (ATAC) (**d**). True positive (Prediction ∩ Ground truth), false positive (Prediction ∉ Ground truth), and false negative (Ground truth ∉ Prediction) predictions for multiplets are highlighted with green, red, and dark blue, respectively. The circled cluster shows the most prominent difference among the prediction results from different methods. COMPOSITE (ADT) almost completely removed the circled cluster, while other methods only removed part of it. Source data are provided as a Source Data file.

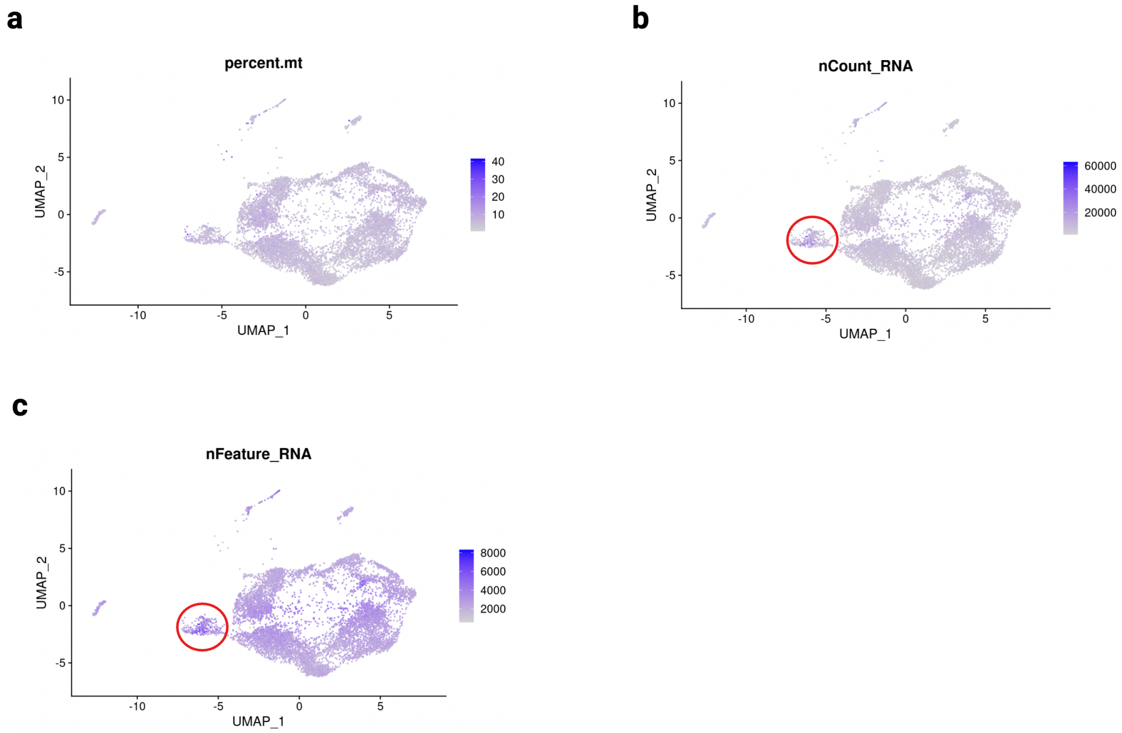

Figure S7: Visualizations of quality control metrics for PB-1 on the weighted nearest neighbors UMAP plots

a-c Visualizations of mitochondrial RNA percentage **(a)**, total unique molecular identifier (UMI) count per droplet **(b)**, and total recorded number of RNA features per droplet **(c)** on the weighted nearest neighbors UMAP generated using all three modalities of data[1]. The ground truth multiplets were removed before generating these plots. The cluster highlighted by the red circles contains many discordant cell types annotated by Azimuth[1] as shown in Figure 5E. The visualizations of quality control metrics indicate that the highlighted cluster exhibits a normal mitochondrial RNA percentage but displays high RNA UMI count and RNA feature numbers. Therefore, it is likely that this cluster comprises multiplets rather than low-quality singlets. Source data are provided as a Source Data file.

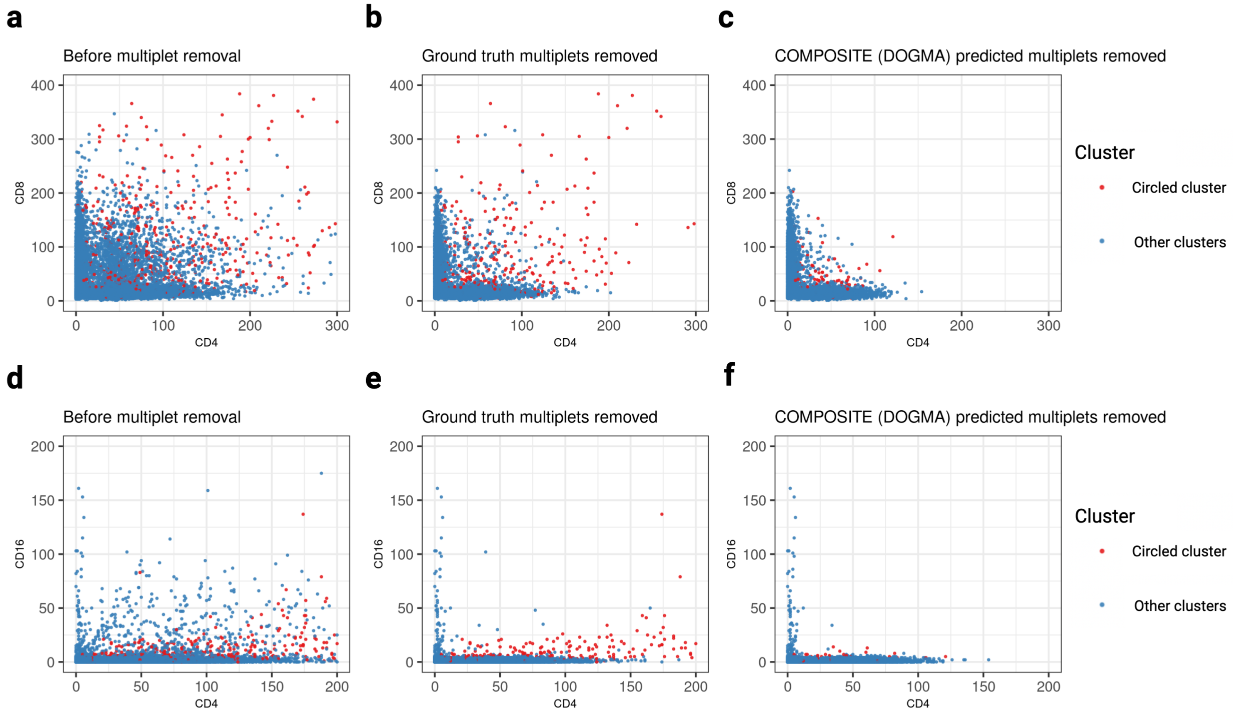

Figure S8: Scatter plots displaying the co-expression of exclusive ADT markers in the PB-1 dataset

a-f Scatter plots depicting the co-expression patterns: between CD4 and CD8 **(a-c)** and between CD4 and CD16 **(d-f)**. Each set of plots represents the following conditions: all droplets prior to multiplet removal **(a & d)**, droplets remaining after the removal of ground truth multiplets **(b & e)**, and droplets remaining after removing COMPOSITE (DOGMA)-predicted multiplets **(c & f)**. The droplets that belong to the circled cluster in **Figure 4a-4e** are highlighted in red. Source data are provided as a Source Data file.

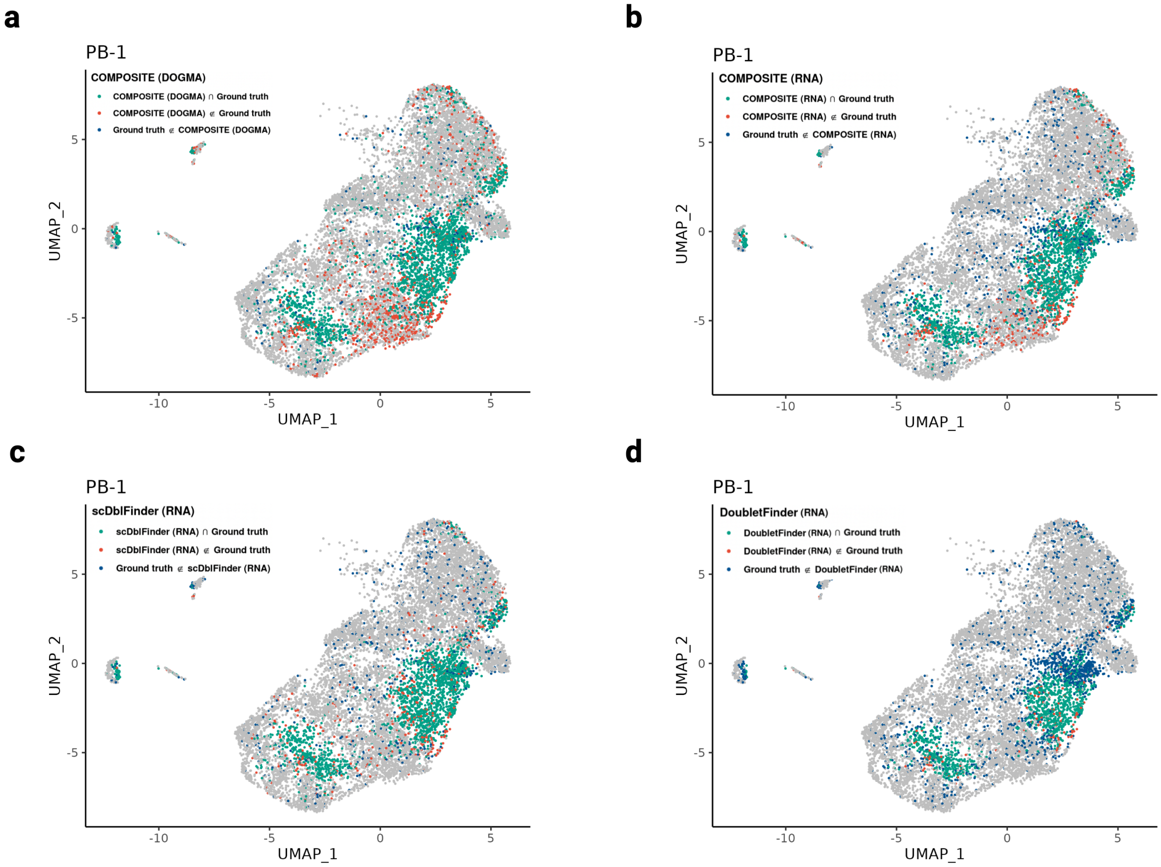

Figure S9: Visualizations of multiplet prediction results on the RNA UMAP (PB-1 dataset)
a-d UMAP plots displaying the comparison between multiplet predictions and ground truth on the PB-1 dataset. The methods shown are COMPOSITE (DOGMA) (**a**), COMPOSITE (RNA) (**b**), scDblFinder (RNA) (**c**), and DoubletFinder (RNA) (**d**). True positive (Prediction ∩ Ground truth), false positive (Prediction ∉ Ground truth), and false negative (Ground truth ∉ Prediction) predictions for multiplets are highlighted with green, red, and dark blue, respectively. The UMAP plots were generated using only the RNA modality of the PB-1 dataset. Source data are provided as a Source Data file.

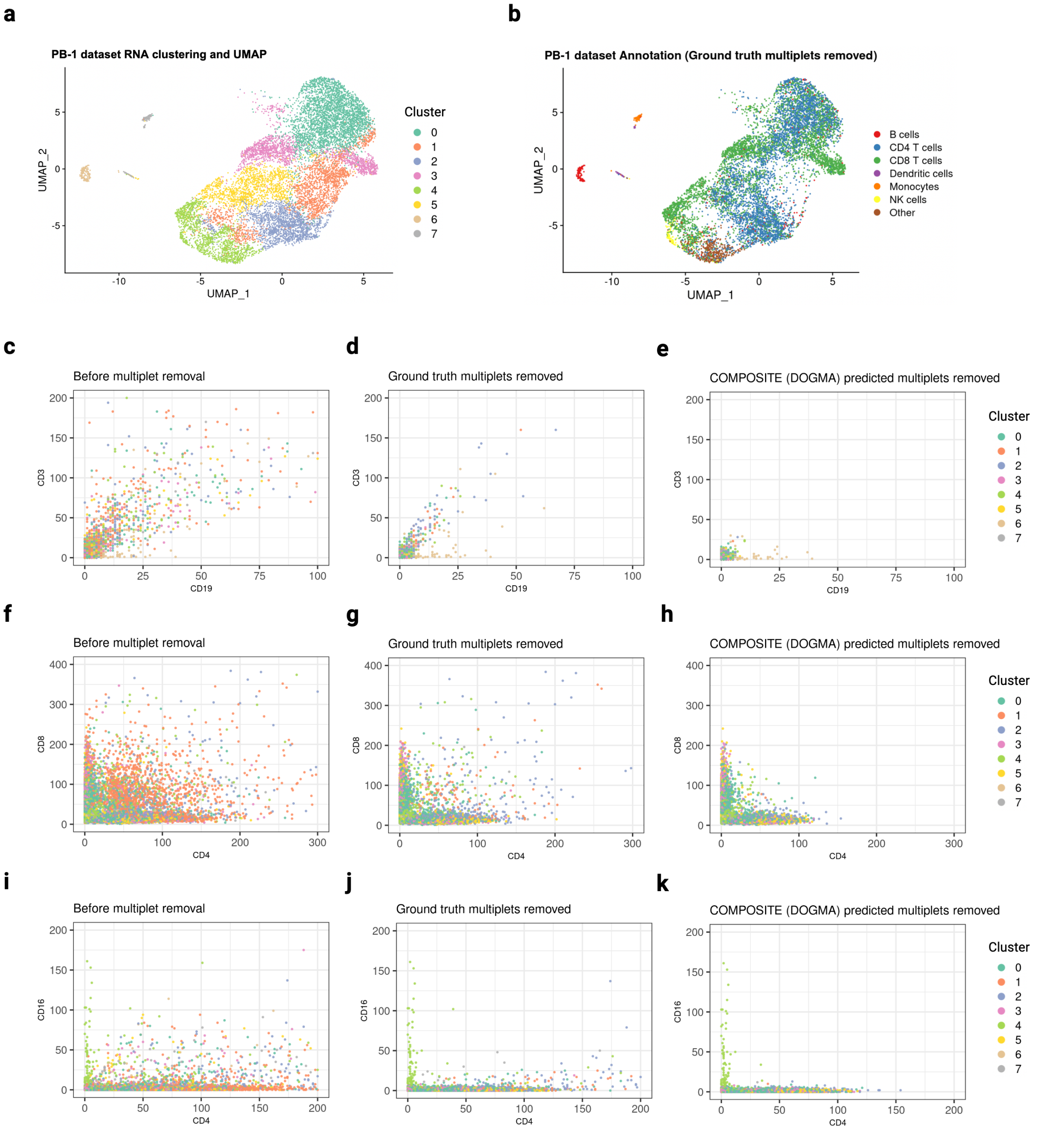

Figure S10: RNA clustering and expression of mutually exclusive ADT markers by cluster in the PB-1 dataset

a Clustering results and UMAP visualization using the RNA modality of the PB-1 dataset
b Azimuth[1] cell type annotation based on the ADT data after removing ground truth multiplets.
c-k Scatter plots depicting the co-expression patterns: between CD19 and CD3 **(c-e)**, between CD4 and CD8 **(f-h)**, and between CD4 and CD16 **(i-k)**. Each set of plots represents the following conditions: all droplets prior to multiplet removal **(c, f & i)**, droplets remaining after the removal of ground truth multiplets **(d, g & j)**, and droplets remaining after removing COMPOSITE (DOGMA) predicted multiplets **(e, h & k)**. The dots are colored by the corresponding cluster identity of the droplet in **a**. Source data are provided as a Source Data file.

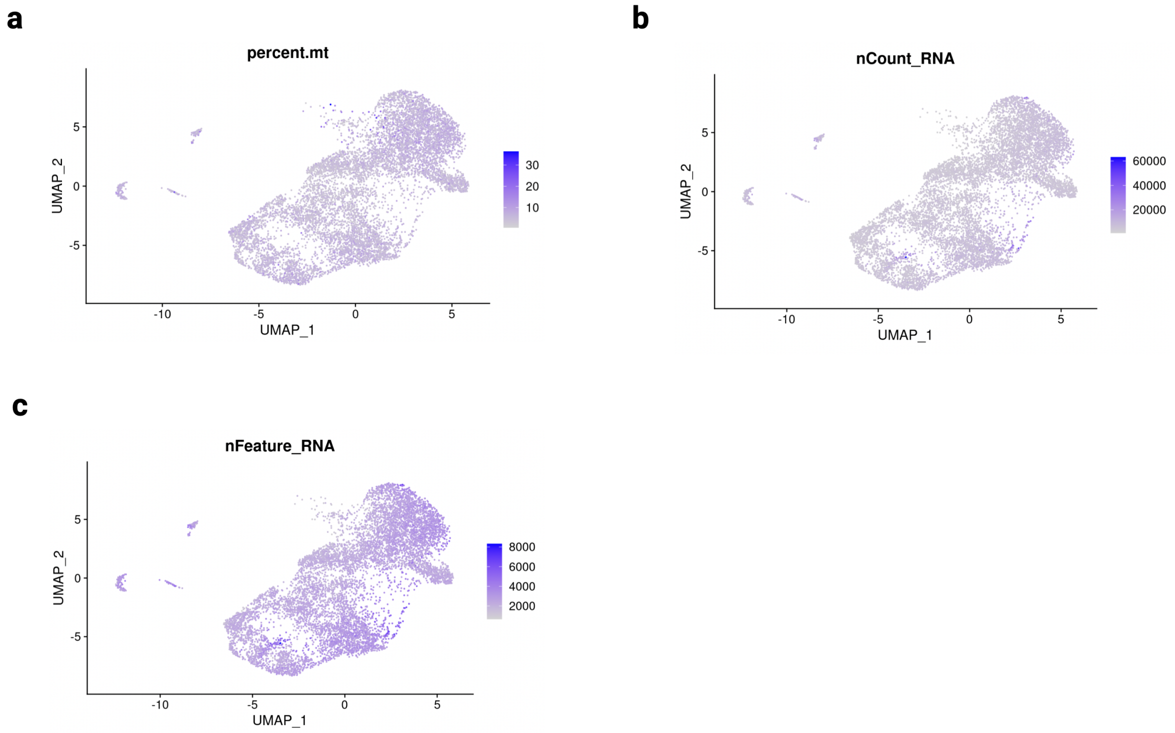

Figure S11: Visualizations of quality control metrics for PB-1 on the RNA UMAP plots

a-c Visualizations of mitochondrial RNA percentage **(a)**, total unique molecular identifier (UMI) count per droplet **(b)**, and total recorded number of RNA features per droplet **(c)** on the UMAP plots generated using the scRNA-seq data. The ground truth multiplets were removed before generating these plots. The droplets with an abnormally high total RNA UMI count and total RNA feature count did not form an obvious cluster. Source data are provided as a Source Data file.

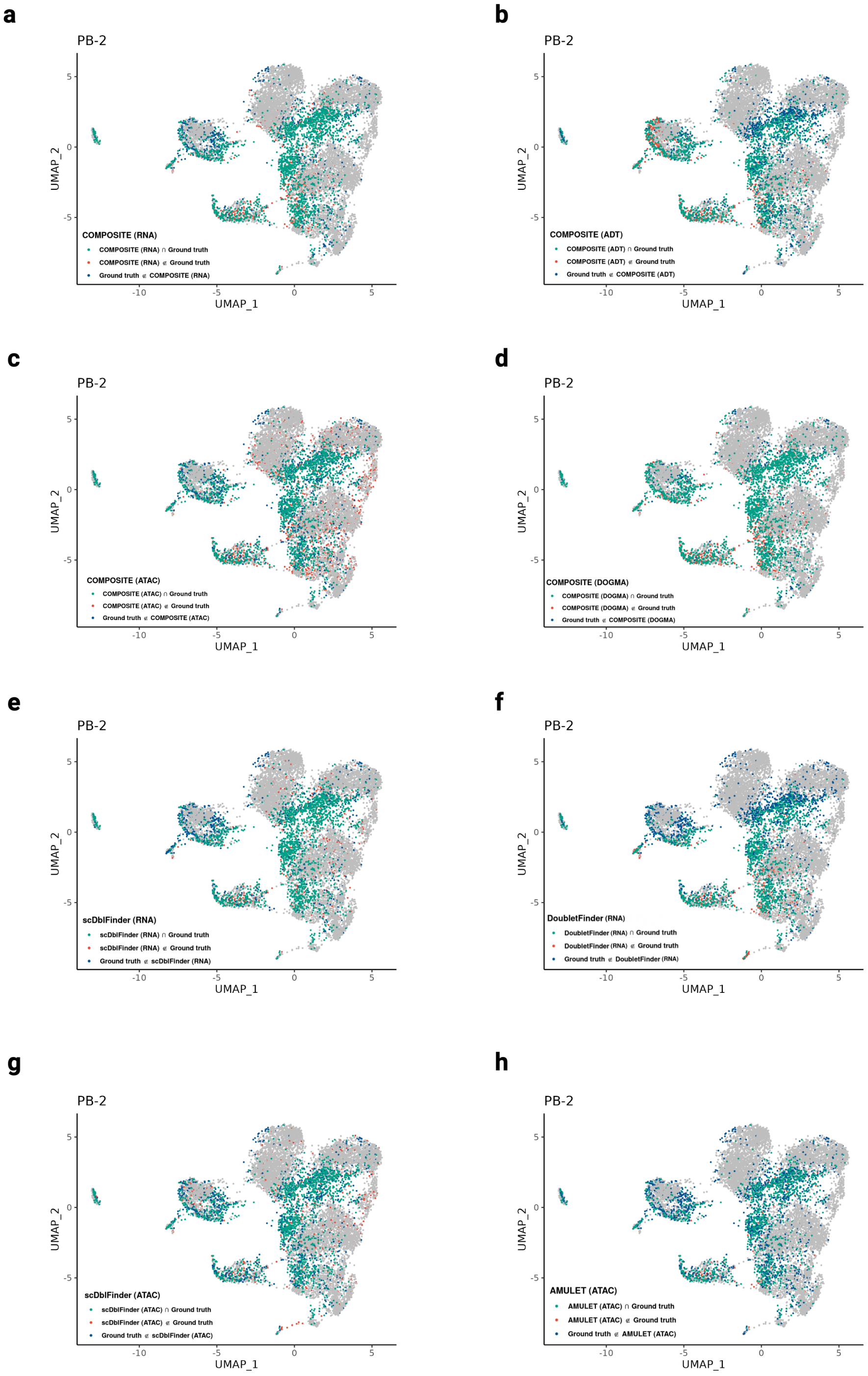

Figure S12: Benchmarking of multiplet prediction methods on the PB-2 dataset
a-h UMAP plots displaying the comparison between multiplet predictions and ground truth on the PB-2 dataset. The methods shown are COMPOSITE (RNA) (**a**), COMPOSITE (ADT) (**b**), COMPOSITE (ATAC) (**c**), COMPOSITE (DOGMA) (**d**), scDblFinder (RNA) (**e**), DoubletFinder (RNA) (**f**), scDblfinder (ATAC) (**g**), and AMULET (ATAC) (**h**). True positive (Prediction ∩ Ground truth), false positive (Prediction ∉ Ground truth), and false negative (Ground truth ∉ Prediction) predictions for multiplets are highlighted with green, red, and dark blue, respectively.

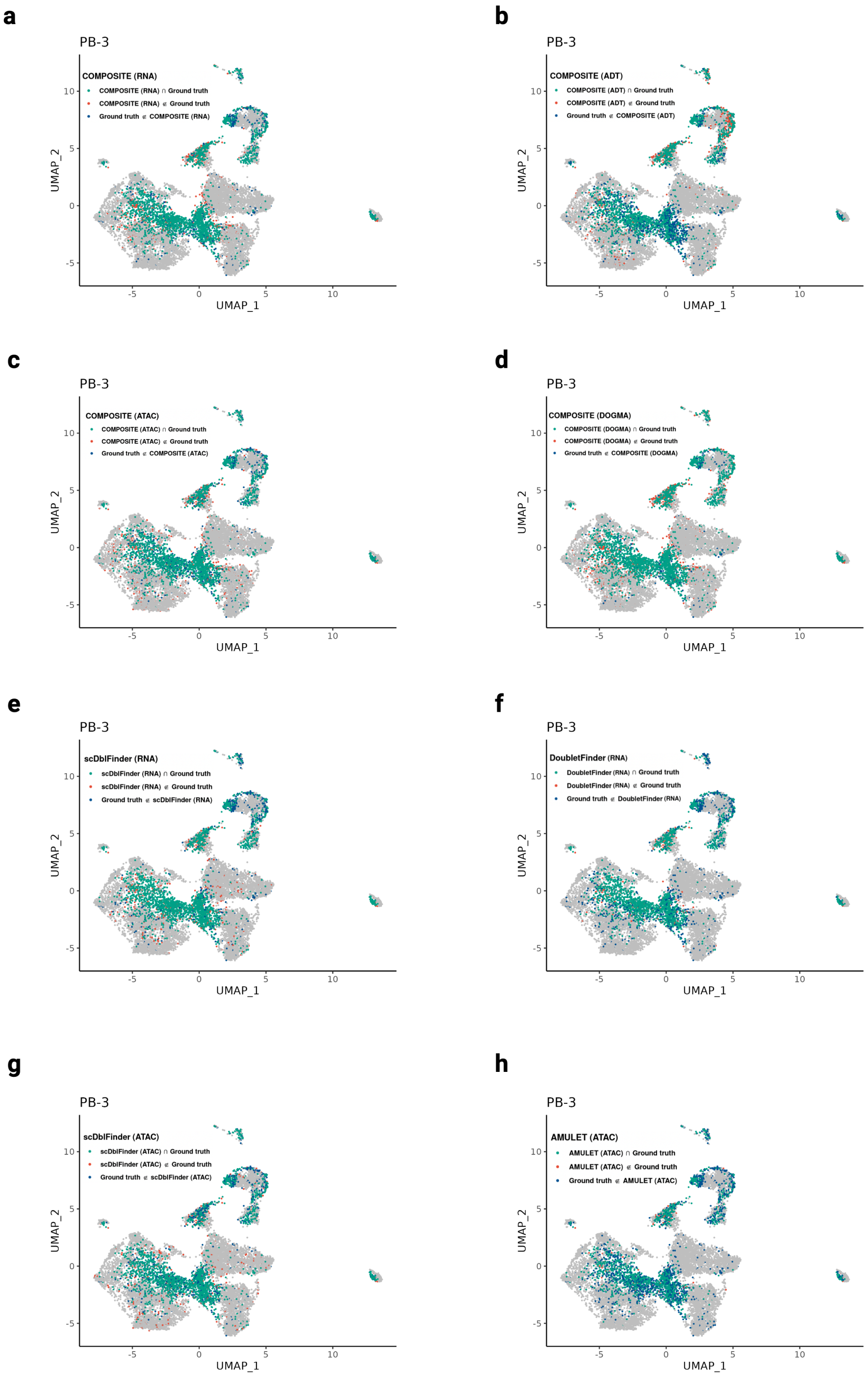

Figure S13: Benchmarking of multiplet prediction methods on the PB-3 dataset

a-h UMAP plots displaying the comparison between multiplet predictions and ground truth on the PB-3 dataset. The methods shown are COMPOSITE (RNA) (**a**), COMPOSITE (ADT) (**b**), COMPOSITE (ATAC) (**c**), COMPOSITE (DOGMA) (**d**), scDblFinder (RNA) (**e**), DoubletFinder (RNA) (**f**), scDblfinder (ATAC) (**g**), and AMULET (ATAC) (**h**). True positive (Prediction ∩ Ground truth), false positive (Prediction ∉ Ground truth), and false negative (Ground truth ∉ Prediction) predictions for multiplets are highlighted with green, red, and dark blue, respectively.

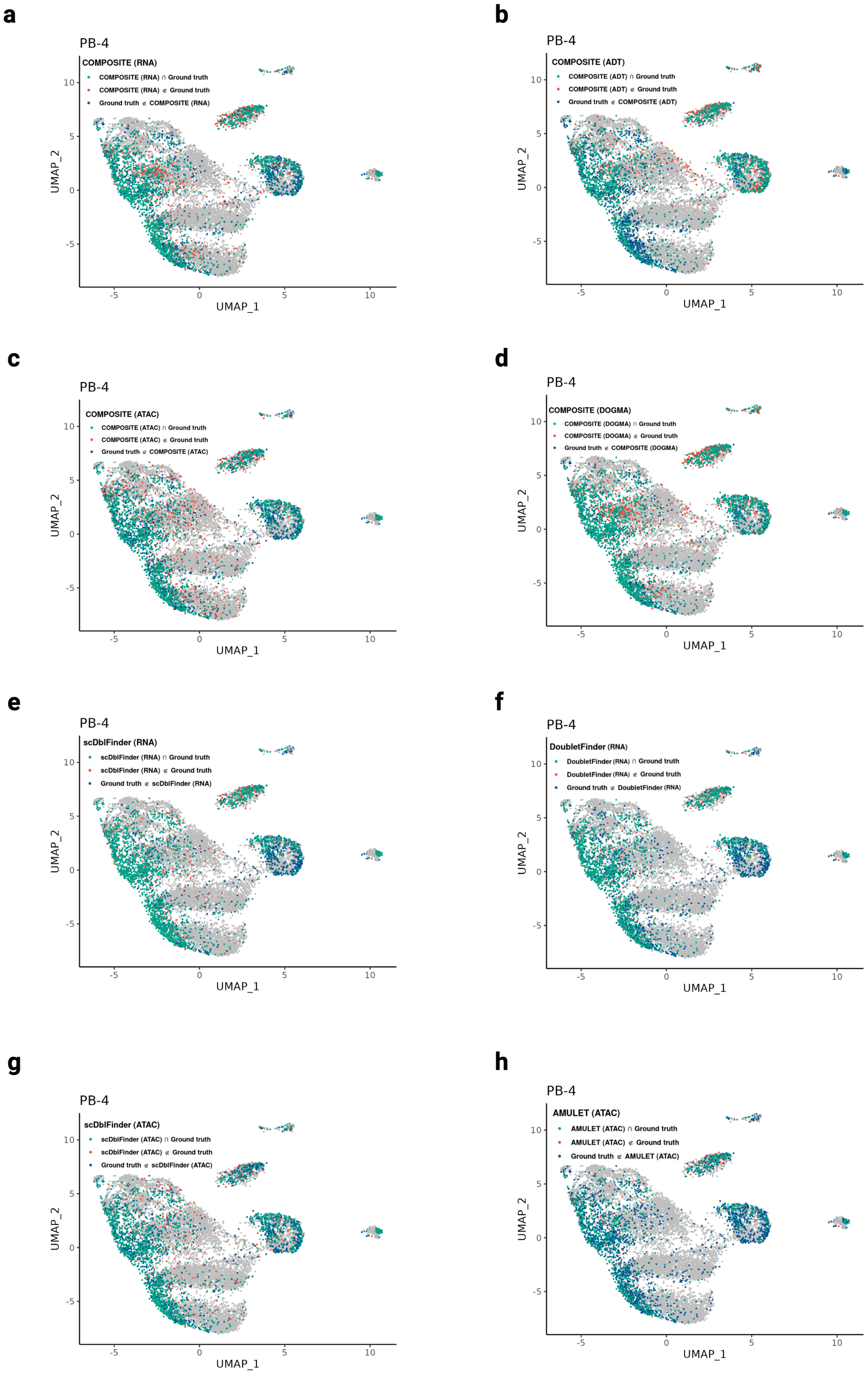

Figure S14: Benchmarking of multiplet prediction methods on the PB-4 dataset

a-h UMAP plots displaying the comparison between multiplet predictions and ground truth on the PB-4 dataset. The methods shown are COMPOSITE (RNA) (**a**), COMPOSITE (ADT) (**b**), COMPOSITE (ATAC) (**c**), COMPOSITE (DOGMA) (**d**), scDblFinder (RNA) (**e**), DoubletFinder (RNA) (**f**), scDblfinder (ATAC) (**g**), and AMULET (ATAC) (**h**). True positive (Prediction ∩ Ground truth), false positive (Prediction ∉ Ground truth), and false negative (Ground truth ∉ Prediction) predictions for multiplets are highlighted with green, red, and dark blue, respectively.

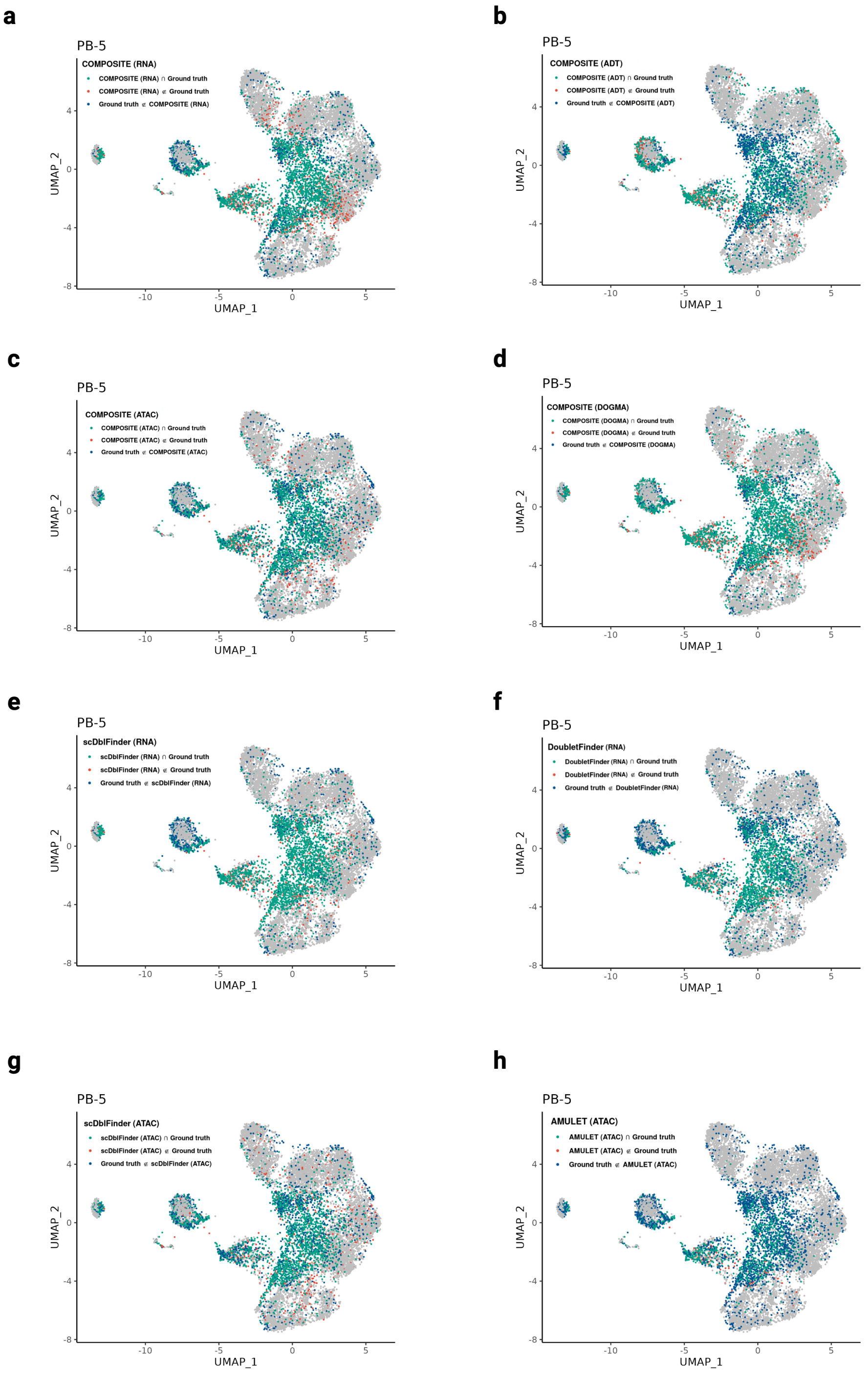

Figure S15: Benchmarking of multiplet prediction methods on the PB-5 dataset

a-h UMAP plots displaying the comparison between multiplet predictions and ground truth on the PB-5 dataset. The methods shown are COMPOSITE (RNA) (**a**), COMPOSITE (ADT) (**b**), COMPOSITE (ATAC) (**c**), COMPOSITE (DOGMA) (**d**), scDblFinder (RNA) (**e**), DoubletFinder (RNA) (**f**), scDblfinder (ATAC) (**g**), and AMULET (ATAC) (**h**). True positive (Prediction ∩ Ground truth), false positive (Prediction ∉ Ground truth), and false negative (Ground truth ∉ Prediction) predictions for multiplets are highlighted with green, red, and dark blue, respectively.

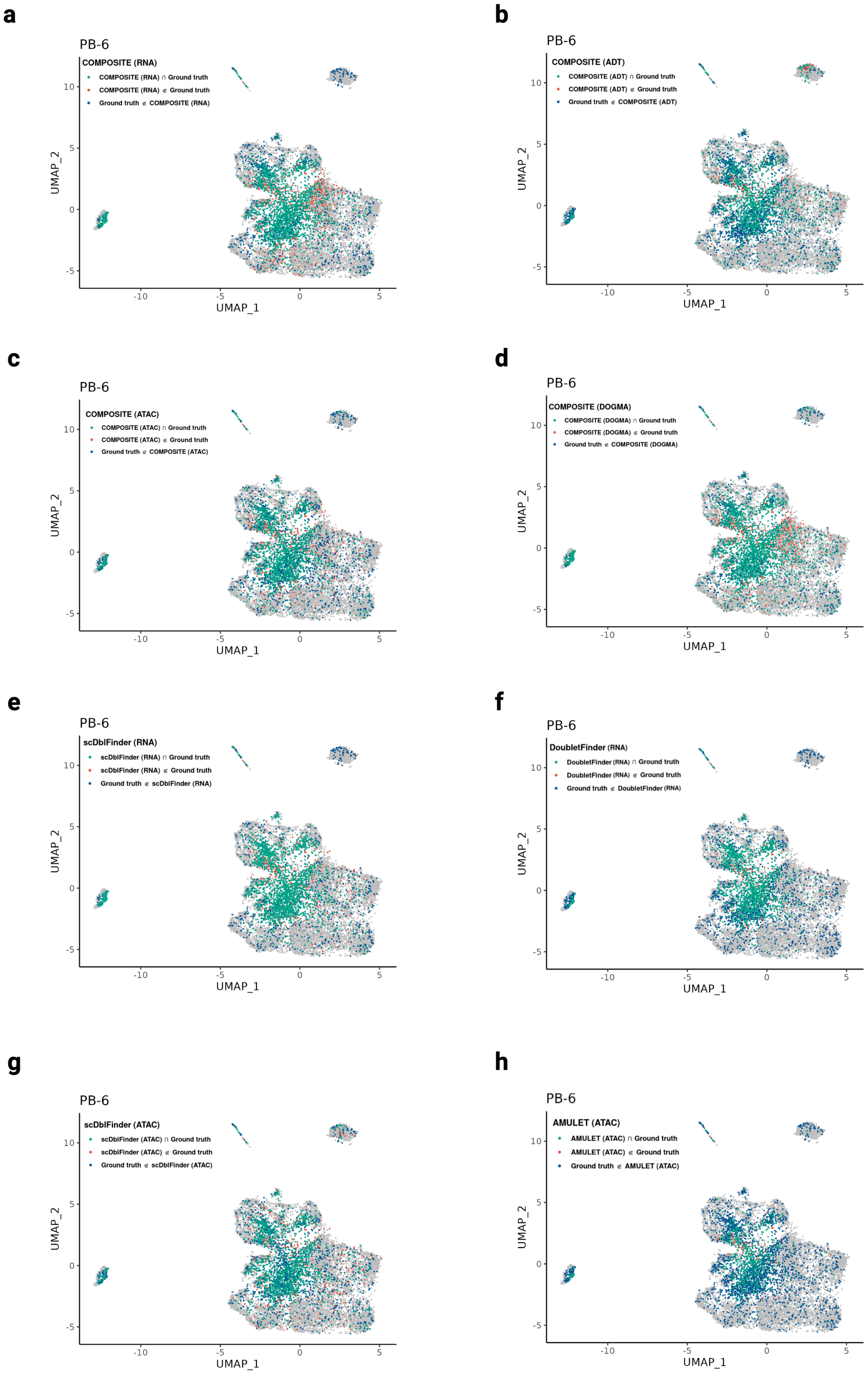

Figure S16: Benchmarking of multiplet prediction methods on the PB-6 dataset

a-h UMAP plots displaying the comparison between multiplet predictions and ground truth on the PB-6 dataset. The methods shown are COMPOSITE (RNA) (**a**), COMPOSITE (ADT) (**b**), COMPOSITE (ATAC) (**c**), COMPOSITE (DOGMA) (**d**), scDblFinder (RNA) (**e**), DoubletFinder (RNA) (**f**), scDblfinder (ATAC) (**g**), and AMULET (ATAC) (**h**). True positive (Prediction ∩ Ground truth), false positive (Prediction ∉ Ground truth), and false negative (Ground truth ∉ Prediction) predictions for multiplets are highlighted with green, red, and dark blue, respectively.

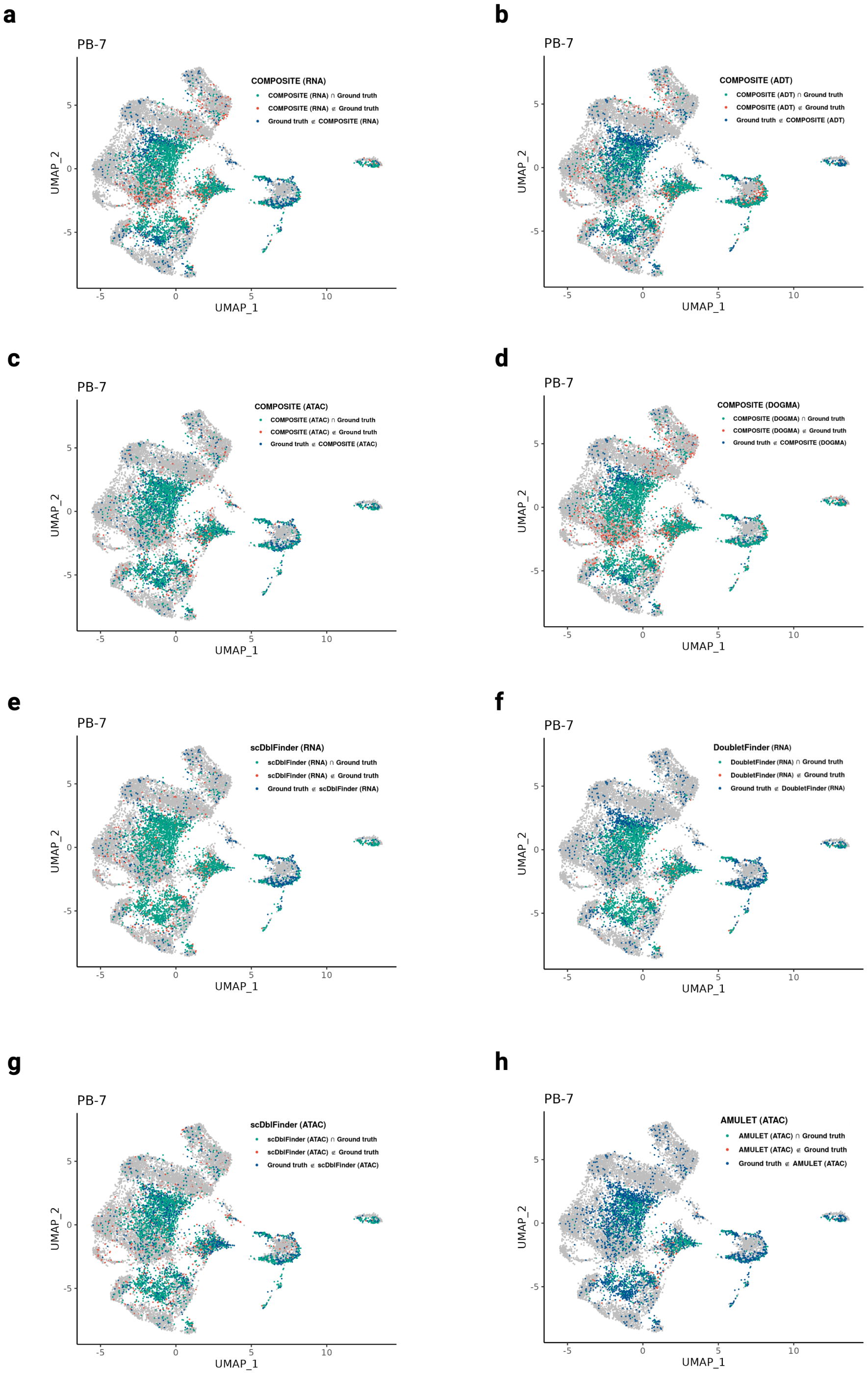

Figure S17: Benchmarking of multiplet prediction methods on the PB-7 dataset

a-h UMAP plots displaying the comparison between multiplet predictions and ground truth on the PB-7 dataset. The methods shown are COMPOSITE (RNA) (**a**), COMPOSITE (ADT) (**b**), COMPOSITE (ATAC) (**c**), COMPOSITE (DOGMA) (**d**), scDblFinder (RNA) (**e**), DoubletFinder (RNA) (**f**), scDblfinder (ATAC) (**g**), and AMULET (ATAC) (**h**). True positive (Prediction ∩ Ground truth), false positive (Prediction ∉ Ground truth), and false negative (Ground truth ∉ Prediction) predictions for multiplets are highlighted with green, red, and dark blue, respectively.

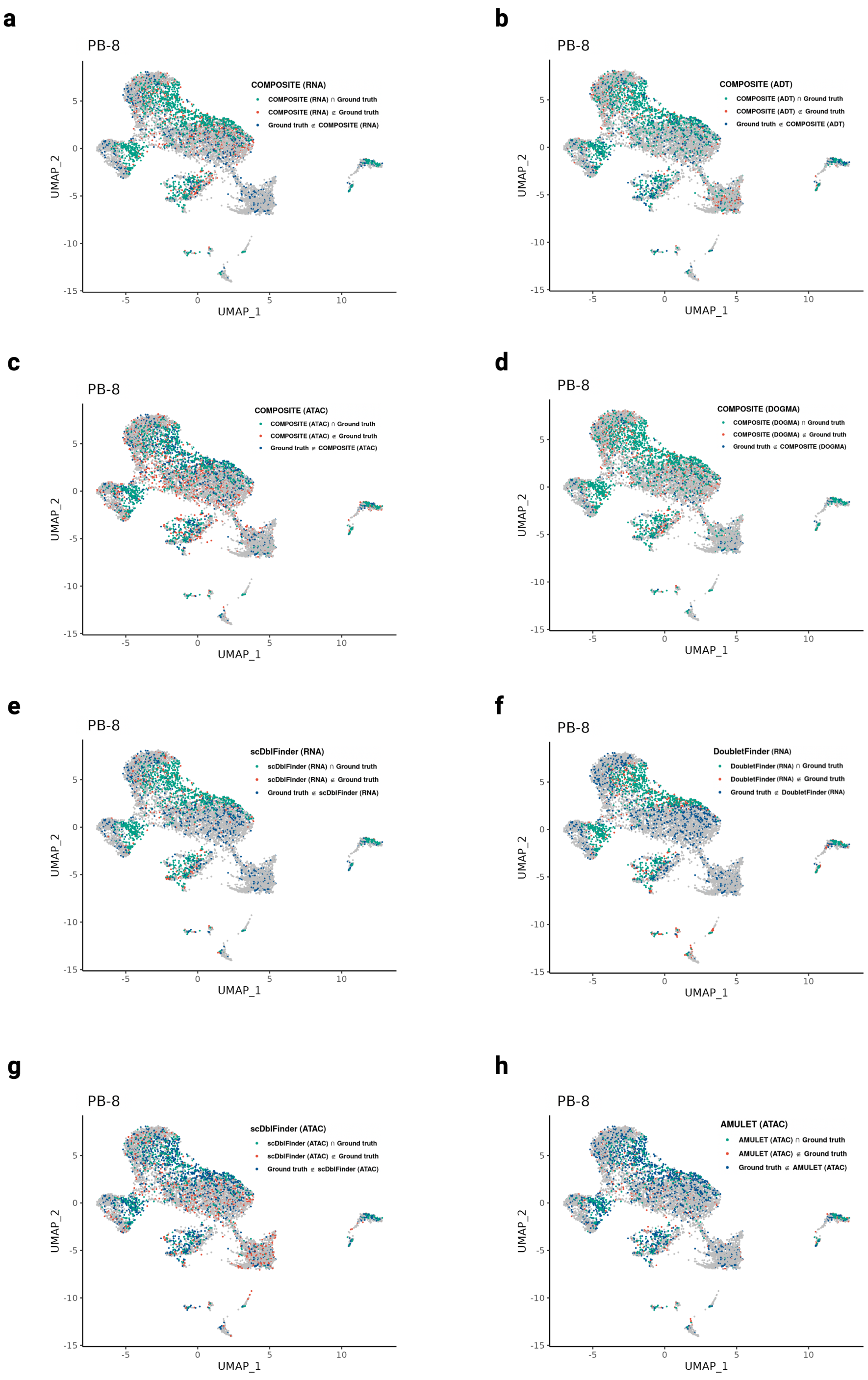

Figure S18: Benchmarking of multiplet prediction methods on the PB-8 dataset

a-h UMAP plots displaying the comparison between multiplet predictions and ground truth on the PB-8 dataset. The methods shown are COMPOSITE (RNA) (**a**), COMPOSITE (ADT) (**b**), COMPOSITE (ATAC) (**c**), COMPOSITE (DOGMA) (**d**), scDblFinder (RNA) (**e**), DoubletFinder (RNA) (**f**), scDblfinder (ATAC) (**g**), and AMULET (ATAC) (**h**). True positive (Prediction ∩ Ground truth), false positive (Prediction ∉ Ground truth), and false negative (Ground truth ∉ Prediction) predictions for multiplets are highlighted with green, red, and dark blue, respectively.

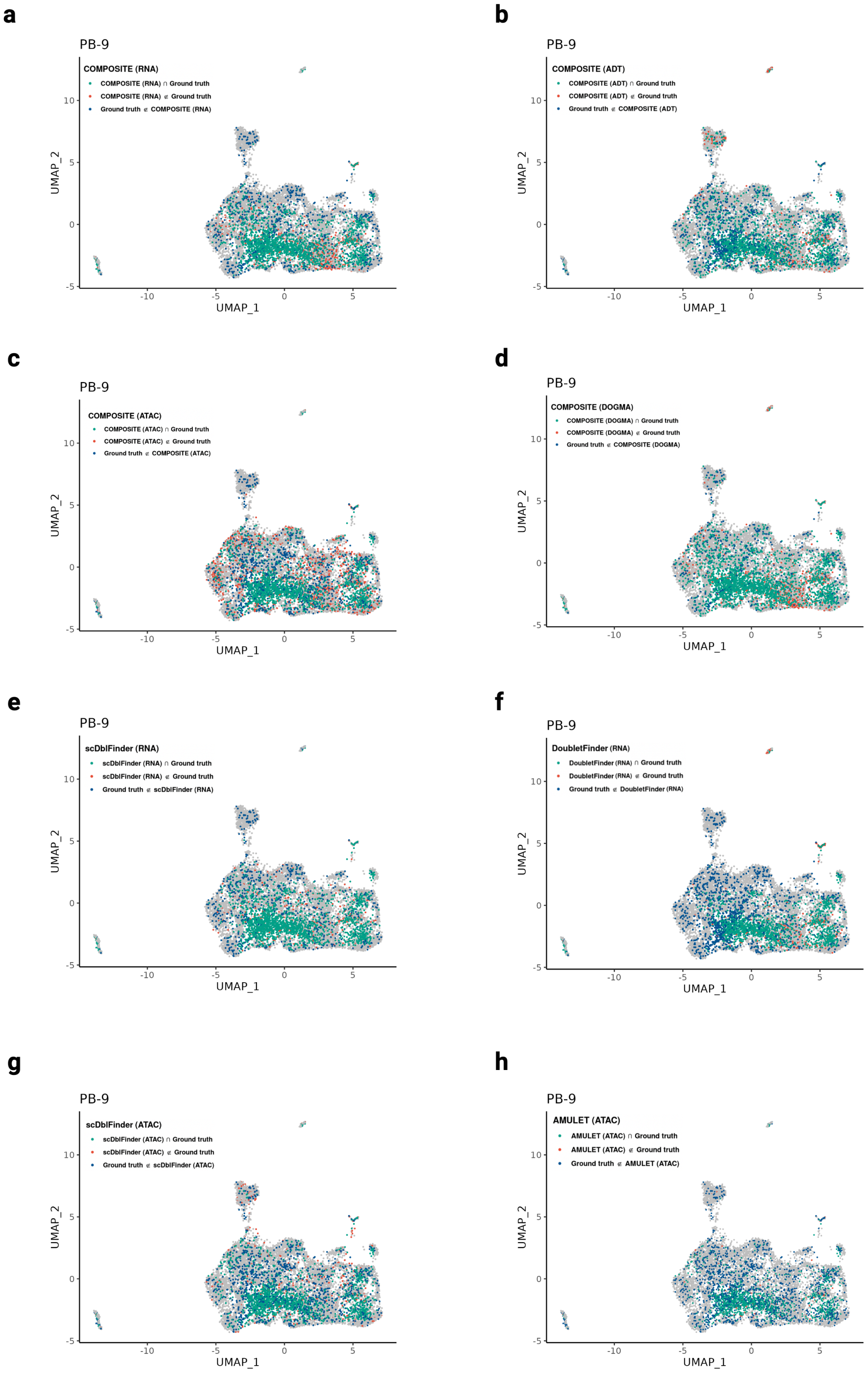

Figure S19: Benchmarking of multiplet prediction methods on the PB-9 dataset

a-h UMAP plots displaying the comparison between multiplet predictions and ground truth on the PB-9 dataset. The methods shown are COMPOSITE (RNA) (**a**), COMPOSITE (ADT) (**b**), COMPOSITE (ATAC) (**c**), COMPOSITE (DOGMA) (**d**), scDblFinder (RNA) (**e**), DoubletFinder (RNA) (**f**), scDblfinder (ATAC) (**g**), and AMULET (ATAC) (**h**). True positive (Prediction ∩ Ground truth), false positive (Prediction ∉ Ground truth), and false negative (Ground truth ∉ Prediction) predictions for multiplets are highlighted with green, red, and dark blue, respectively.

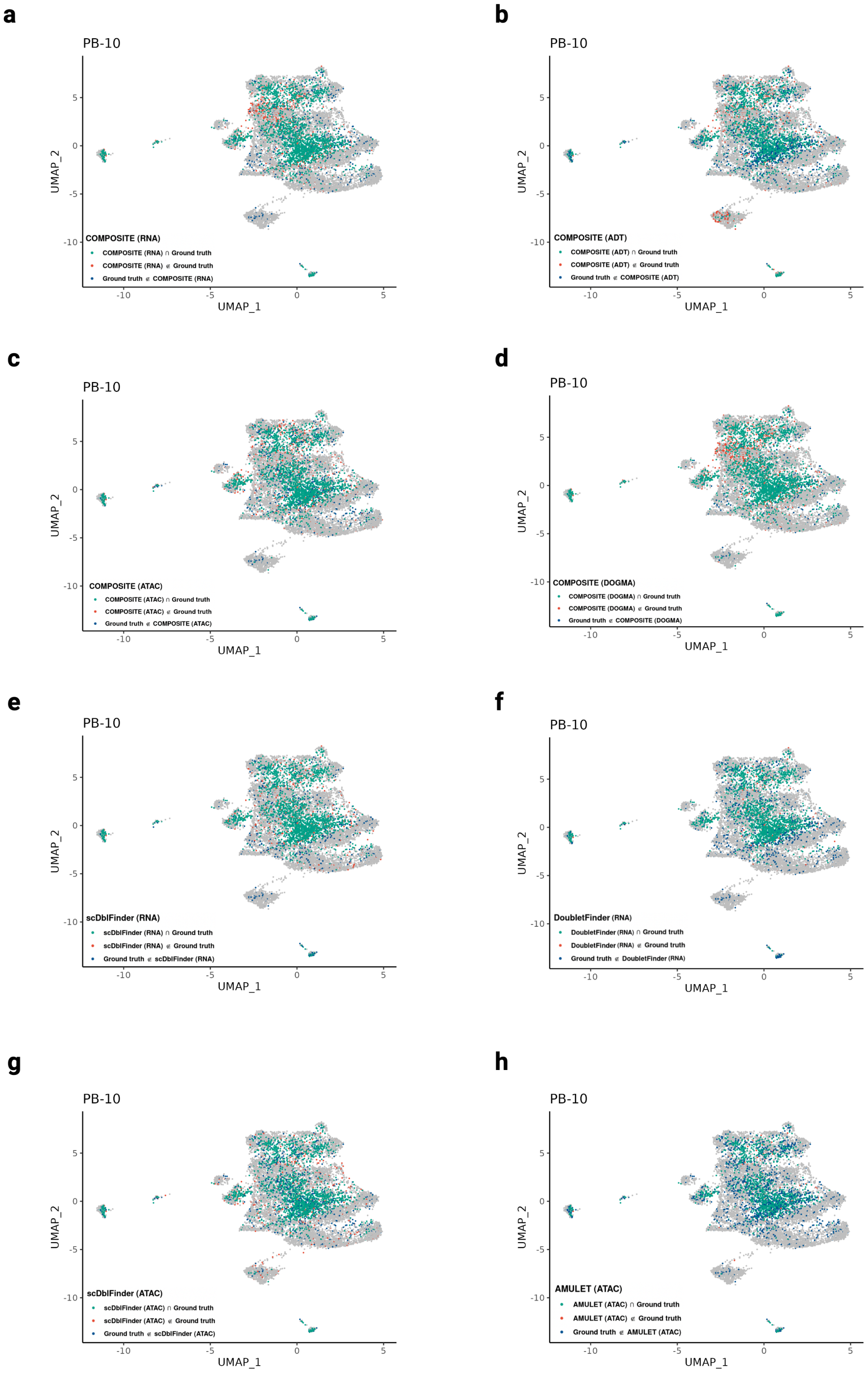

Figure S20: Benchmarking of multiplet prediction methods on the PB-10 dataset

a-h UMAP plots displaying the comparison between multiplet predictions and ground truth on the PB-10 dataset. The methods shown are COMPOSITE (RNA) (**a**), COMPOSITE (ADT) (**b**), COMPOSITE (ATAC) (**c**), COMPOSITE (DOGMA) (**d**), scDblFinder (RNA) (**e**), DoubletFinder (RNA) (**f**), scDblfinder (ATAC) (**g**), and AMULET (ATAC) (**h**). True positive (Prediction ∩ Ground truth), false positive (Prediction ∉ Ground truth), and false negative (Ground truth ∉ Prediction) predictions for multiplets are highlighted with green, red, and dark blue, respectively.

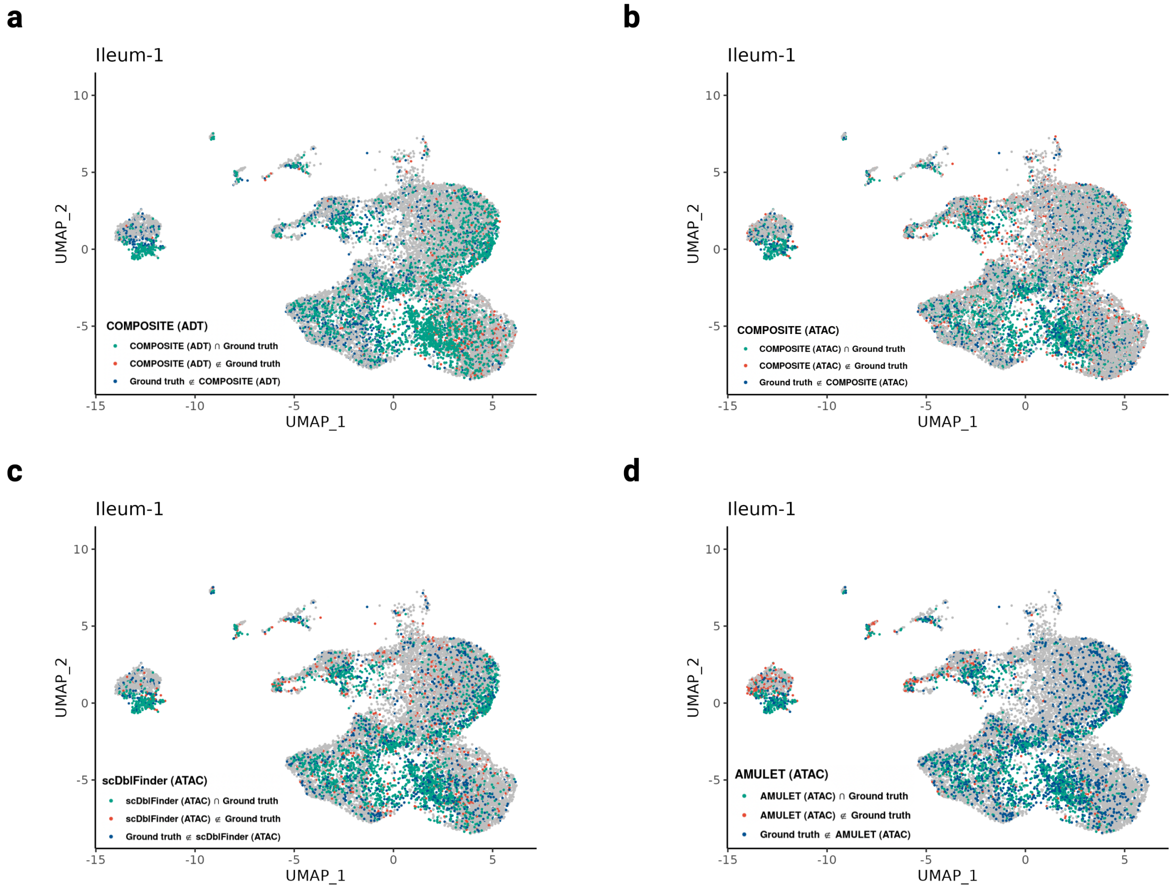

Figure S21: Supplementary benchmarking of multiplet prediction methods on the Ileum-1 dataset

a-d: UMAP plots displaying the comparison between multiplet predictions and ground truth on the Ileum-1 dataset. The methods shown are COMPOSITE (ADT) (**a**), COMPOSITE (ATAC) (**b**), scDblFinder (ATAC) (**c**), and AMULET (ATAC) (**d**). True positive (Prediction ∩ Ground truth), false positive (Prediction ∉ Ground truth), and false negative (Ground truth ∉ Prediction) predictions for multiplets are highlighted with green, red, and dark blue, respectively.

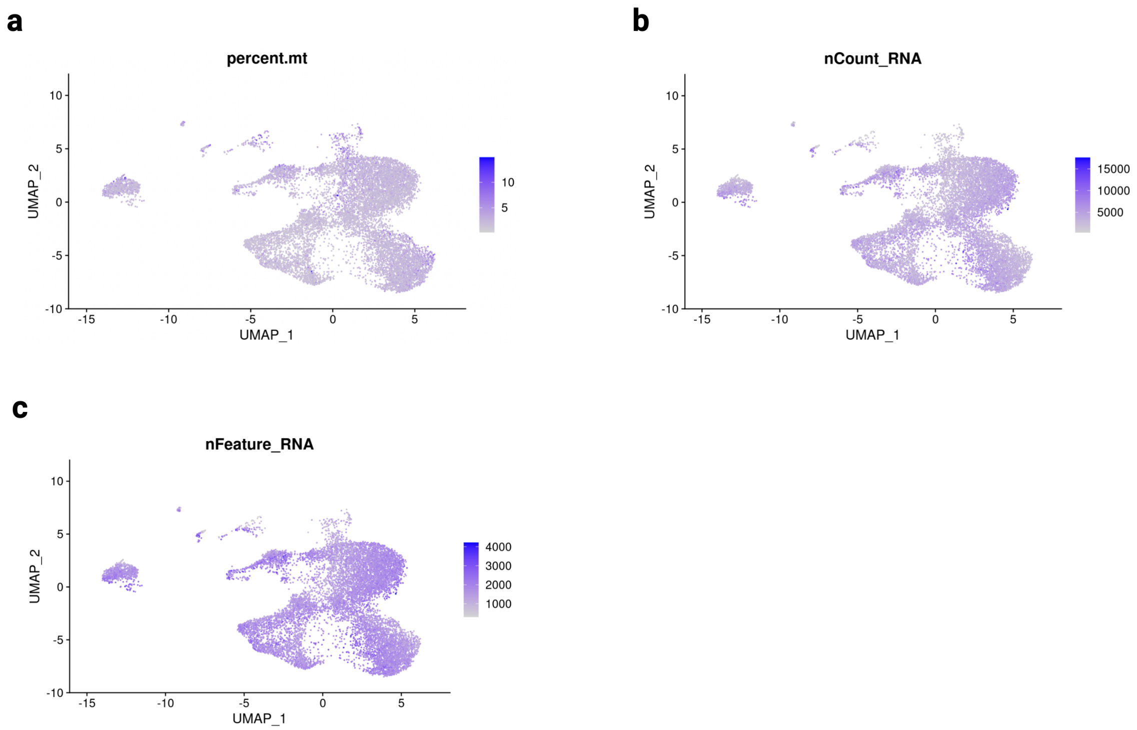

Figure S22: Visualizations of quality control metrics for Ileum-1 on the UMAP plots
Visualizations of mitochondrial RNA percentage **(a)**, total UMI count per droplet **(b)**, and total recorded number of RNA features per droplet **(c)** on the weighted nearest neighbors UMAP generated using all three modalities of data[1]. The ground truth multiplets have been removed.

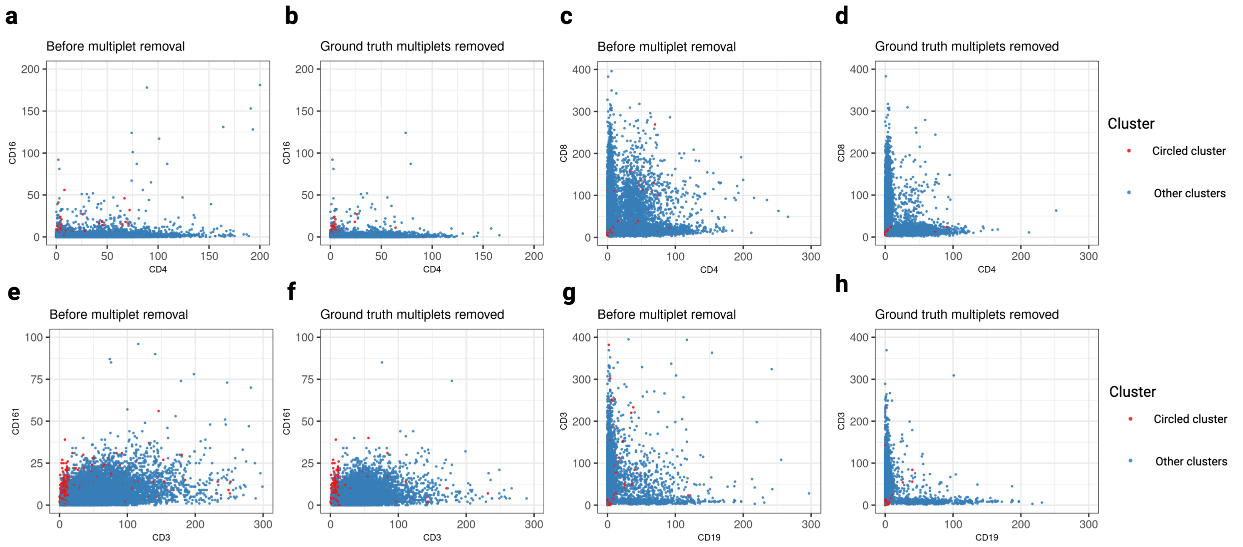

Figure S23: Scatter plots displaying the co-expression of exclusive ADT markers in the Ileum-1 dataset

Scatter plots depicting the co-expression patterns: **(a-b)** between CD4 and CD16, **(c-d)** between CD4 and CD8, **(e-f)** between CD3 and CD161, and **(g-h)** between CD19 and CD3. Each set of plots represents the following conditions: **(a, c, e, & g)** all droplets prior to multiplet removal, and **(b, d, f, & h)** droplets left after removing ground truth multiplets. The droplets that belong to the circled cluster in **Figure 5a-5d** are highlighted in red.

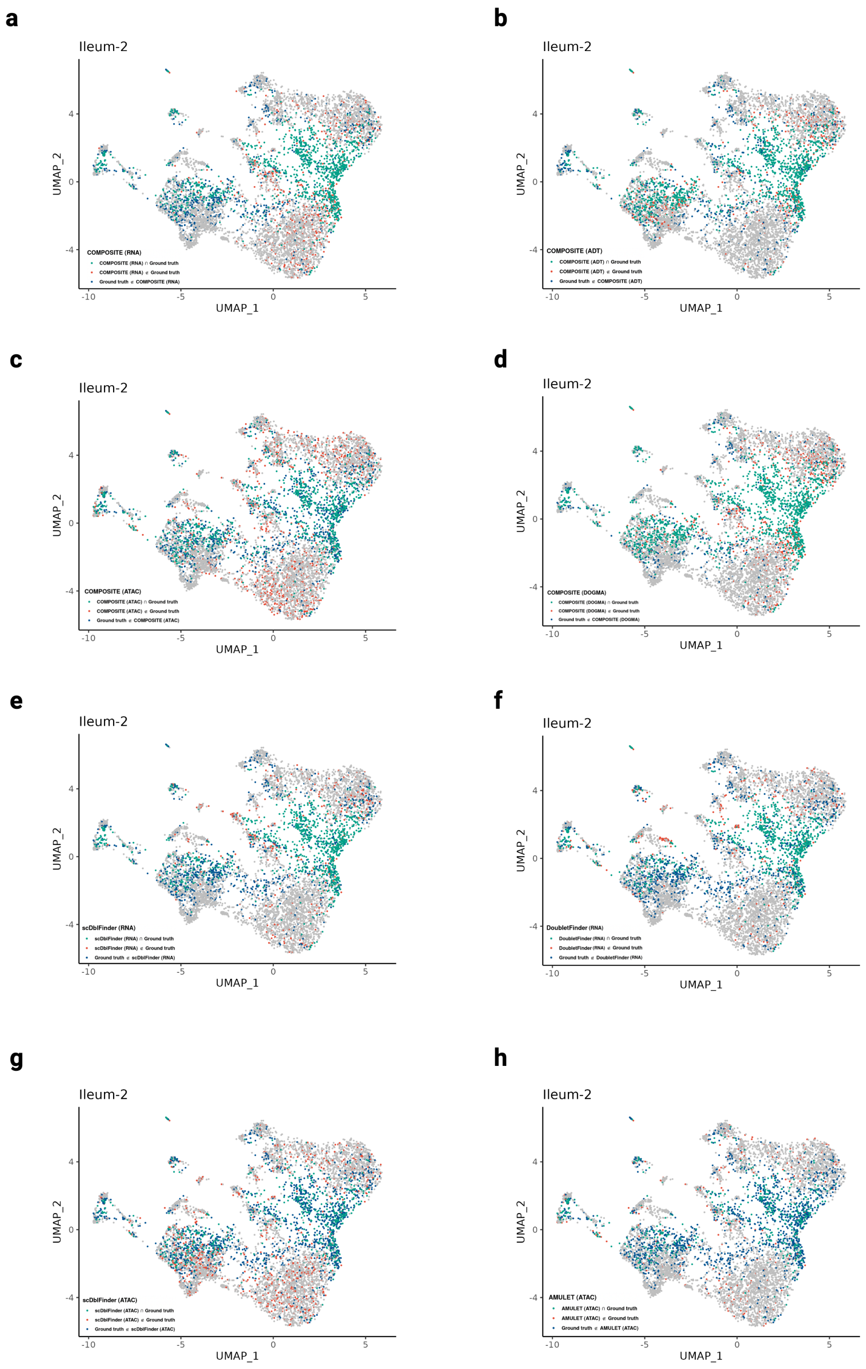

Figure S24: Benchmarking of multiplet prediction methods on the Ileum-2 dataset

a-h UMAP plots displaying the comparison between multiplet predictions and ground truth on the Ileum-2 dataset. The methods shown are COMPOSITE (RNA) (**a**), COMPOSITE (ADT) (**b**), COMPOSITE (ATAC) (**c**), COMPOSITE (DOGMA) (**d**), scDblFinder (RNA) (**e**), DoubletFinder (RNA) (**f**), scDblfinder (ATAC) (**g**), and AMULET (ATAC) (**h**). True positive (Prediction ∩ Ground truth), false positive (Prediction ∉ Ground truth), and false negative (Ground truth ∉ Prediction) predictions for multiplets are highlighted with green, red, and dark blue, respectively.

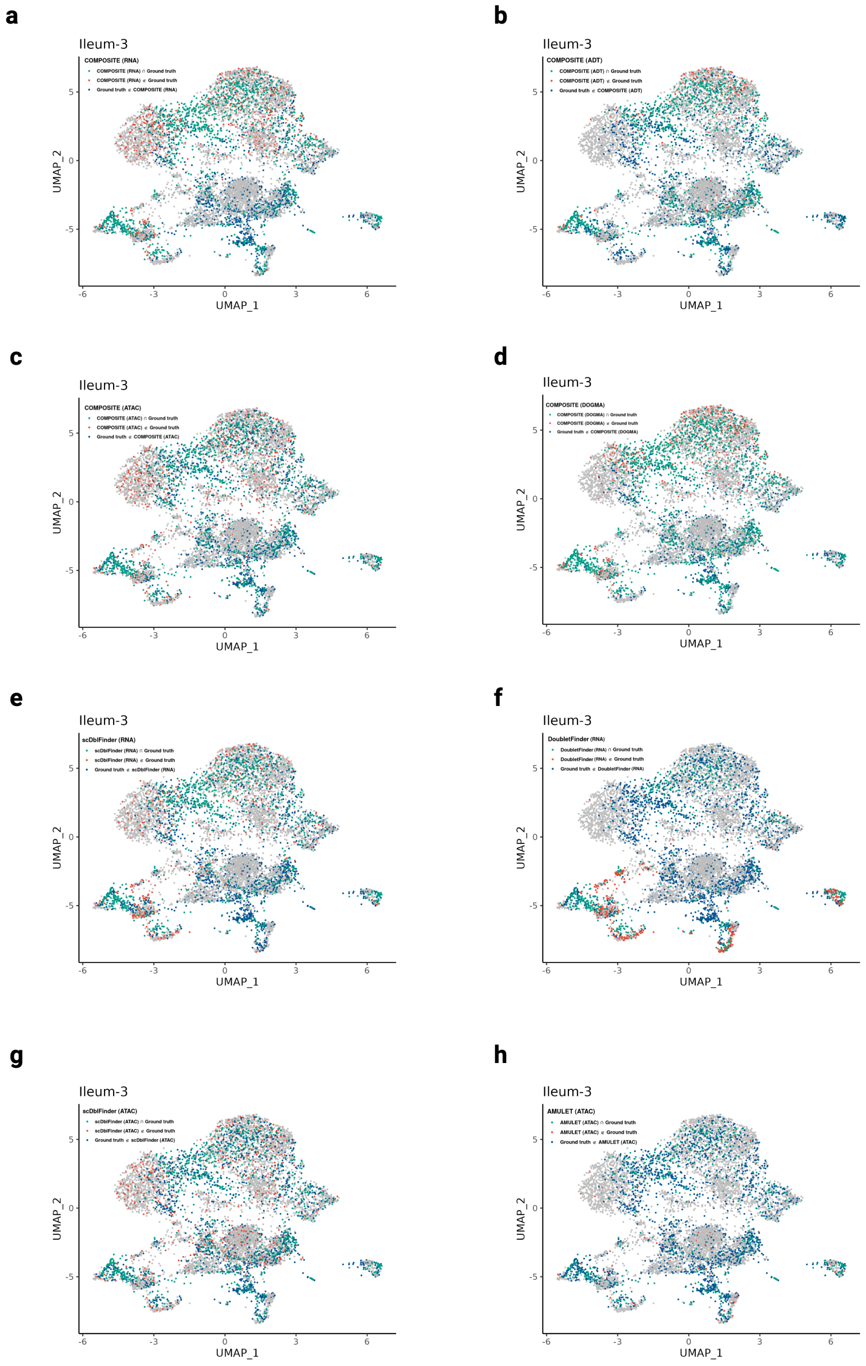

Figure S25: Benchmarking of multiplet prediction methods on the Ileum-3 dataset

a-h UMAP plots displaying the comparison between multiplet predictions and ground truth on the Ileum-3 dataset. The methods shown are COMPOSITE (RNA) (**a**), COMPOSITE (ADT) (**b**), COMPOSITE (ATAC) (**c**), COMPOSITE (DOGMA) (**d**), scDblFinder (RNA) (**e**), DoubletFinder (RNA) (**f**), scDblfinder (ATAC) (**g**), and AMULET (ATAC) (**h**). True positive (Prediction ∩ Ground truth), false positive (Prediction ∉ Ground truth), and false negative (Ground truth ∉ Prediction) predictions for multiplets are highlighted with green, red, and dark blue, respectively.

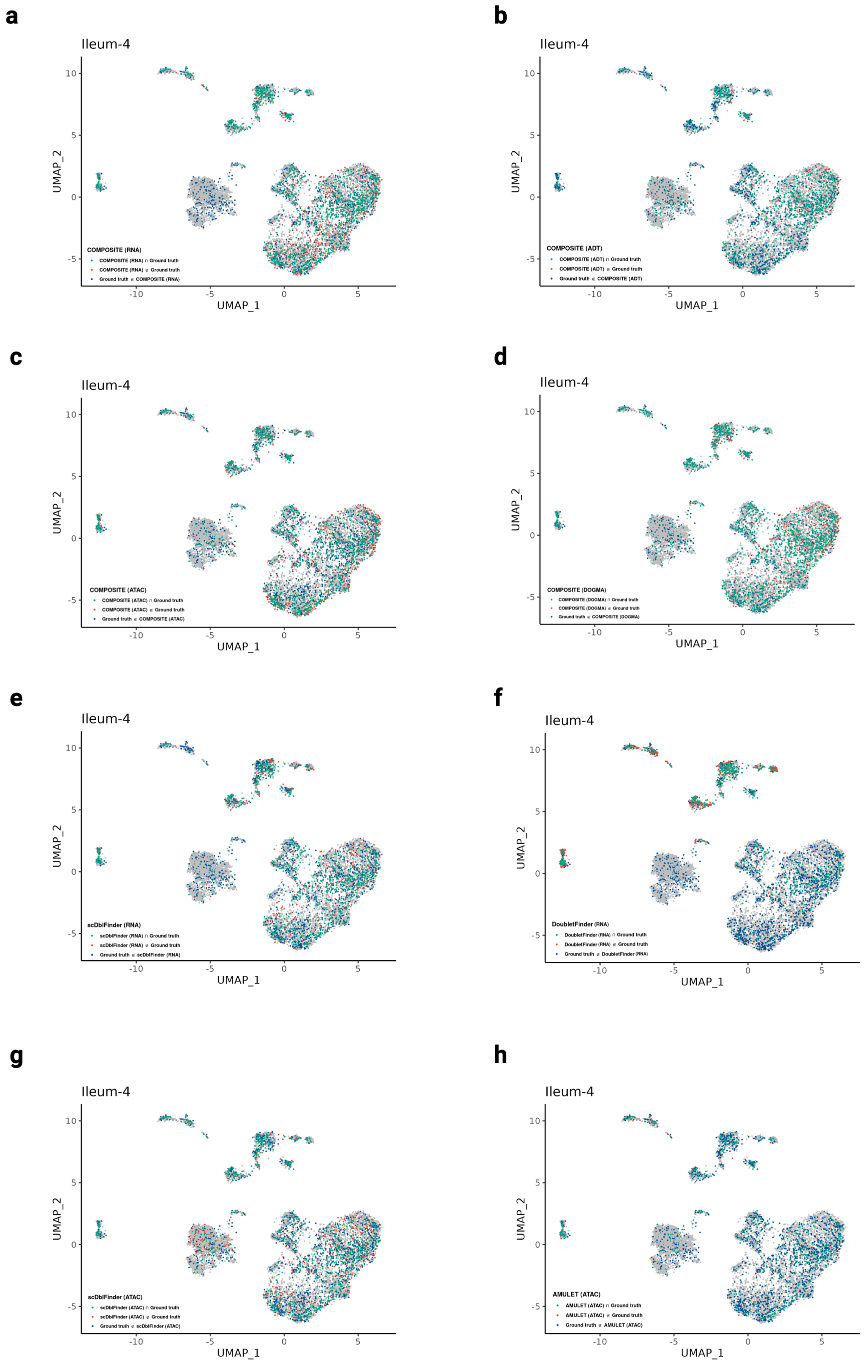

Figure S26: Benchmarking of multiplet prediction methods on the Ileum-4 dataset

a-h UMAP plots displaying the comparison between multiplet predictions and ground truth on the Ileum-4 dataset. The methods shown are COMPOSITE (RNA) (**a**), COMPOSITE (ADT) (**b**), COMPOSITE (ATAC) (**c**), COMPOSITE (DOGMA) (**d**), scDblFinder (RNA) (**e**), DoubletFinder (RNA) (**f**), scDblfinder (ATAC) (**g**), and AMULET (ATAC) (**h**). True positive (Prediction ∩ Ground truth), false positive (Prediction ∉ Ground truth), and false negative (Ground truth ∉ Prediction) predictions for multiplets are highlighted with green, red, and dark blue, respectively.

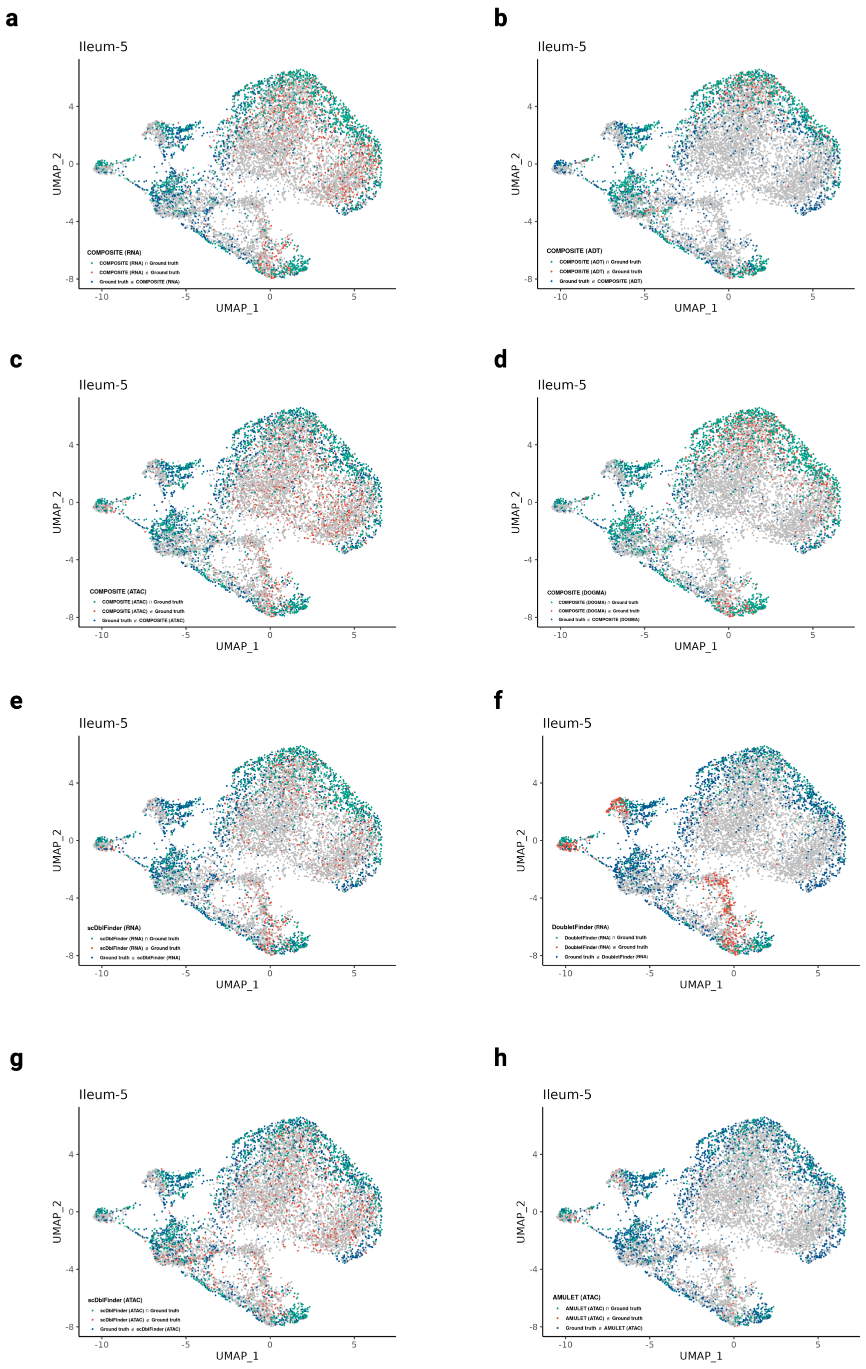

Figure S27: Benchmarking of multiplet prediction methods on the Ileum-5 dataset

a-h UMAP plots displaying the comparison between multiplet predictions and ground truth on the Ileum-5 dataset. The methods shown are COMPOSITE (RNA) (**a**), COMPOSITE (ADT) (**b**), COMPOSITE (ATAC) (**c**), COMPOSITE (DOGMA) (**d**), scDblFinder (RNA) (**e**), DoubletFinder (RNA) (**f**), scDblfinder (ATAC) (**g**), and AMULET (ATAC) (**h**). True positive (Prediction ∩ Ground truth), false positive (Prediction ∉ Ground truth), and false negative (Ground truth ∉ Prediction) predictions for multiplets are highlighted with green, red, and dark blue, respectively.

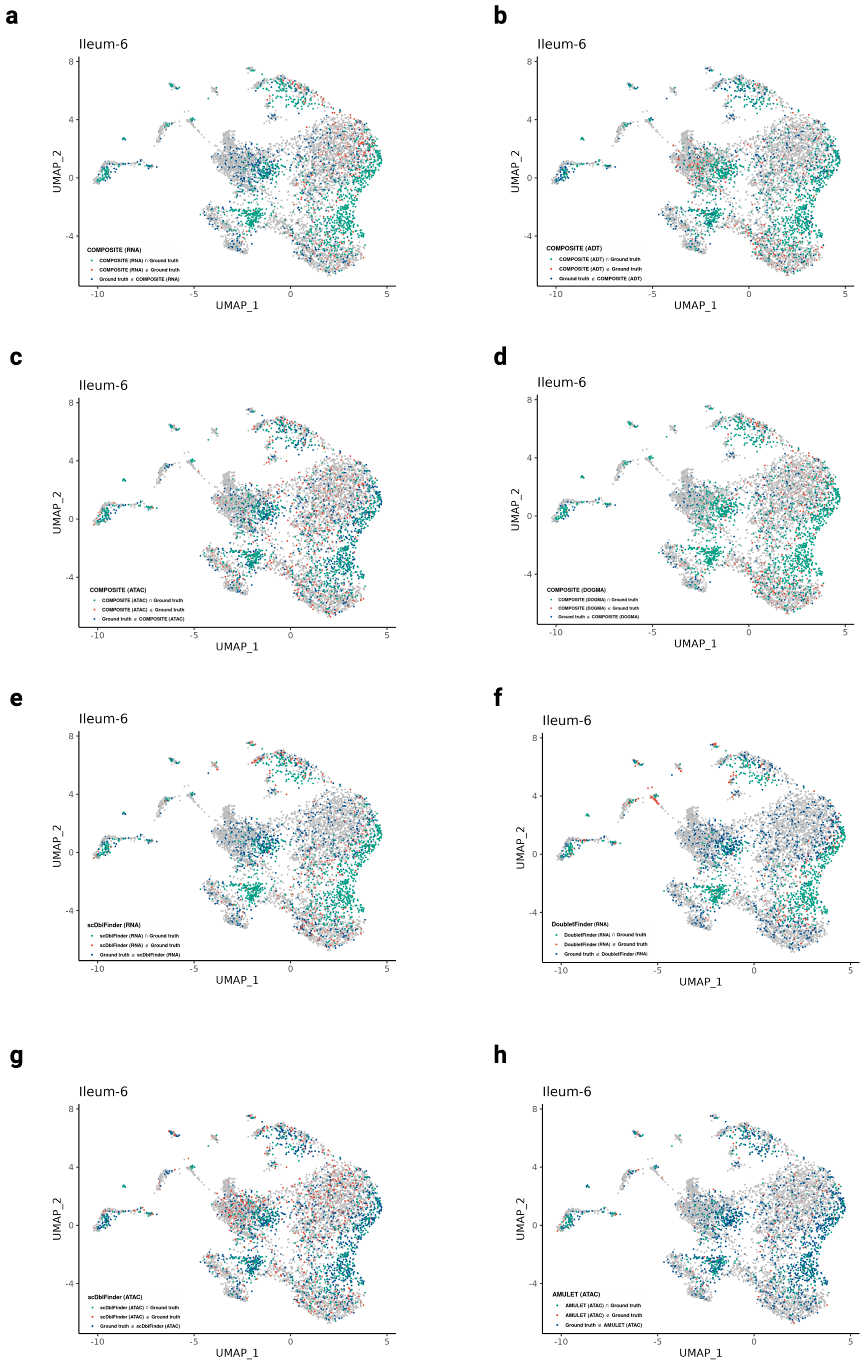

Figure S28: Benchmarking of multiplet prediction methods on the Ileum-6 dataset

a-h UMAP plots displaying the comparison between multiplet predictions and ground truth on the Ileum-6 dataset. The methods shown are COMPOSITE (RNA) (**a**), COMPOSITE (ADT) (**b**), COMPOSITE (ATAC) (**c**), COMPOSITE (DOGMA) (**d**), scDblFinder (RNA) (**e**), DoubletFinder (RNA) (**f**), scDblfinder (ATAC) (**g**), and AMULET (ATAC) (**h**). True positive (Prediction ∩ Ground truth), false positive (Prediction ∉ Ground truth), and false negative (Ground truth ∉ Prediction) predictions for multiplets are highlighted with green, red, and dark blue, respectively.

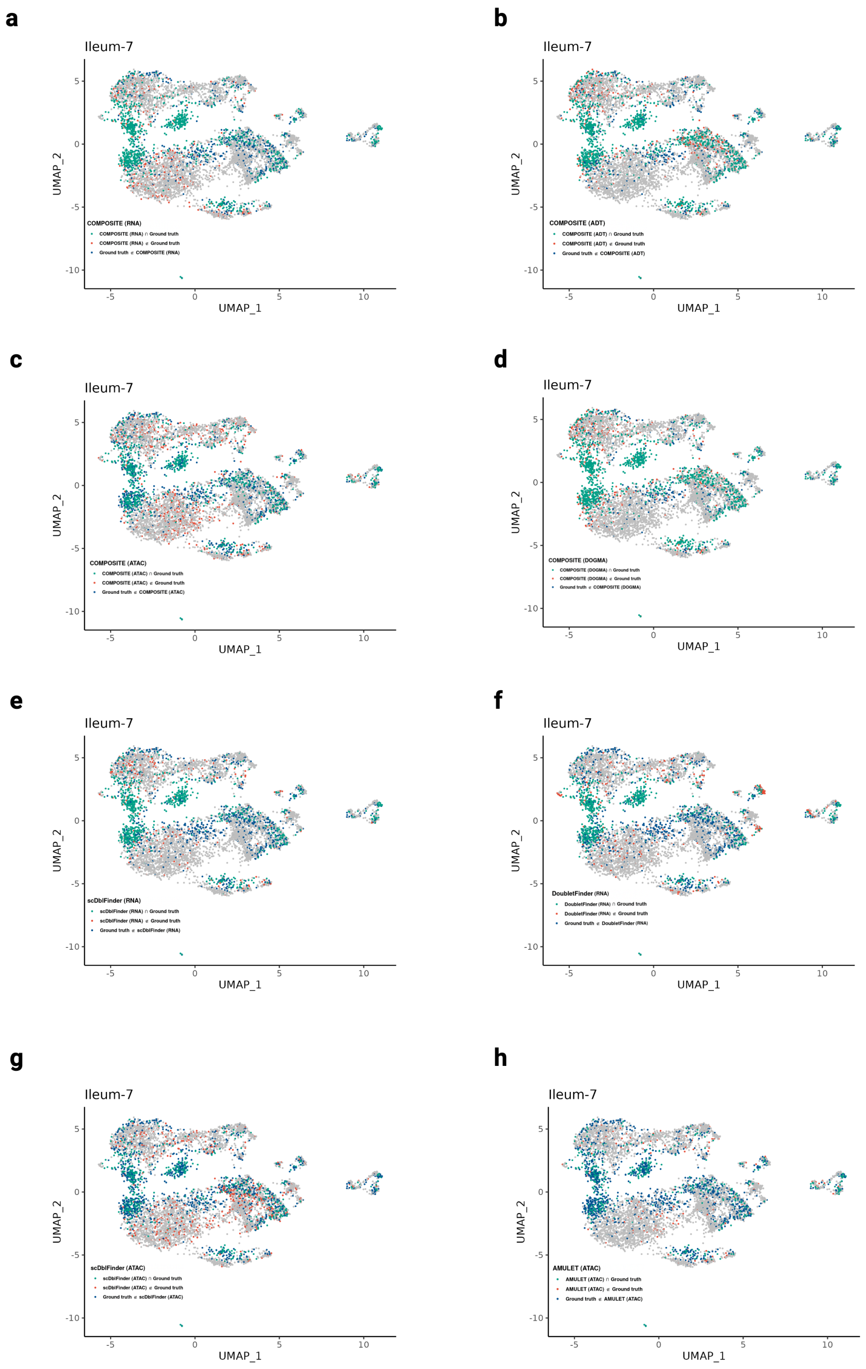

Figure S29: Benchmarking of multiplet prediction methods on the Ileum-7 dataset

a-h UMAP plots displaying the comparison between multiplet predictions and ground truth on the Ileum-7 dataset. The methods shown are COMPOSITE (RNA) (**a**), COMPOSITE (ADT) (**b**), COMPOSITE (ATAC) (**c**), COMPOSITE (DOGMA) (**d**), scDblFinder (RNA) (**e**), DoubletFinder (RNA) (**f**), scDblfinder (ATAC) (**g**), and AMULET (ATAC) (**h**). True positive (Prediction ∩ Ground truth), false positive (Prediction ∉ Ground truth), and false negative (Ground truth ∉ Prediction) predictions for multiplets are highlighted with green, red, and dark blue, respectively.

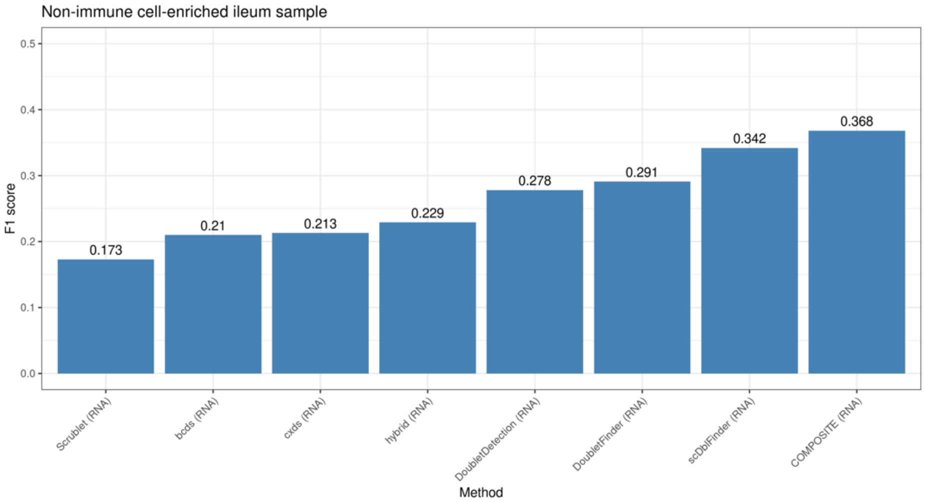

Figure S30: Benchmarking of multiplet detection performance (in terms of F1 score) on the non-immune cell-enriched colon scRNA-seq dataset

Source data are provided as a Source Data file.

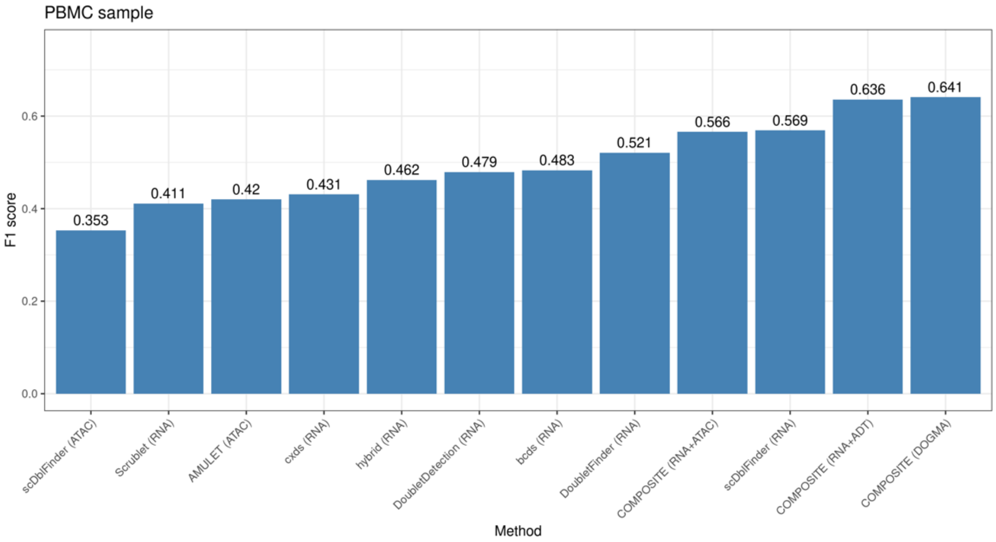

Figure S31: Benchmarking of multiplet detection performance (in terms of F1 score) on the PBMC DOGMA-seq dataset

Source data are provided as a Source Data file.

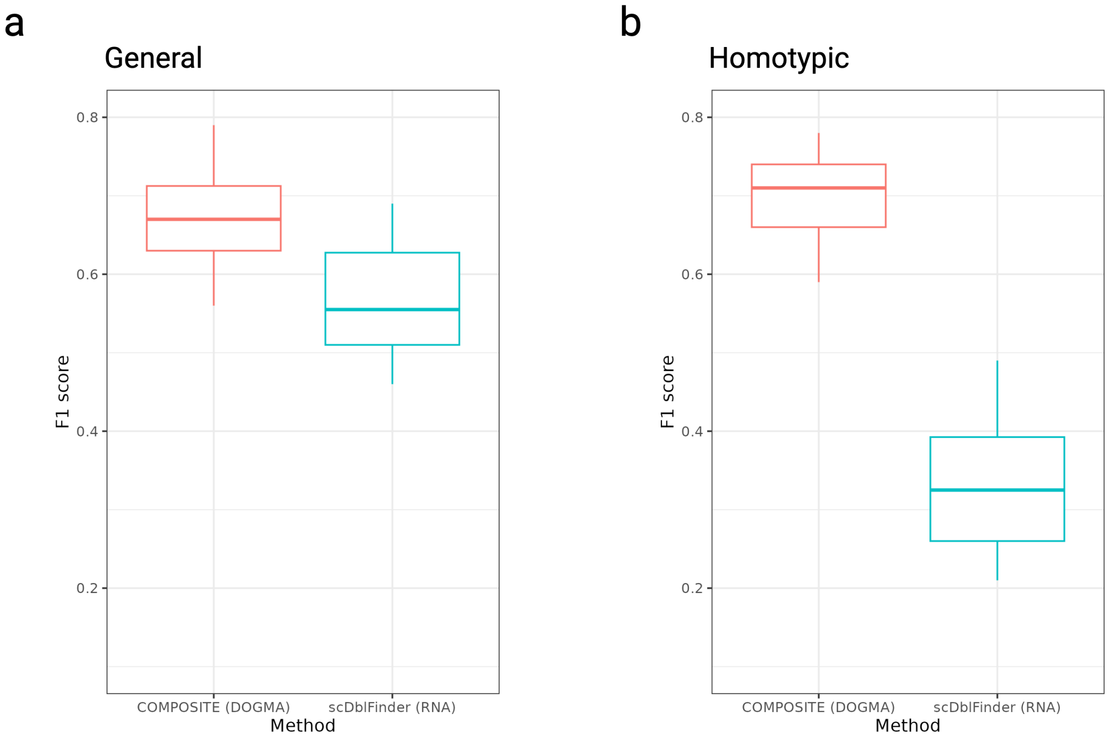

Figure S32: Simulation results comparing COMPOSITE (DOGMA) and scDblFinder (RNA) in terms of their abilities to detect general doublets (a) and homotypic doublets (b).

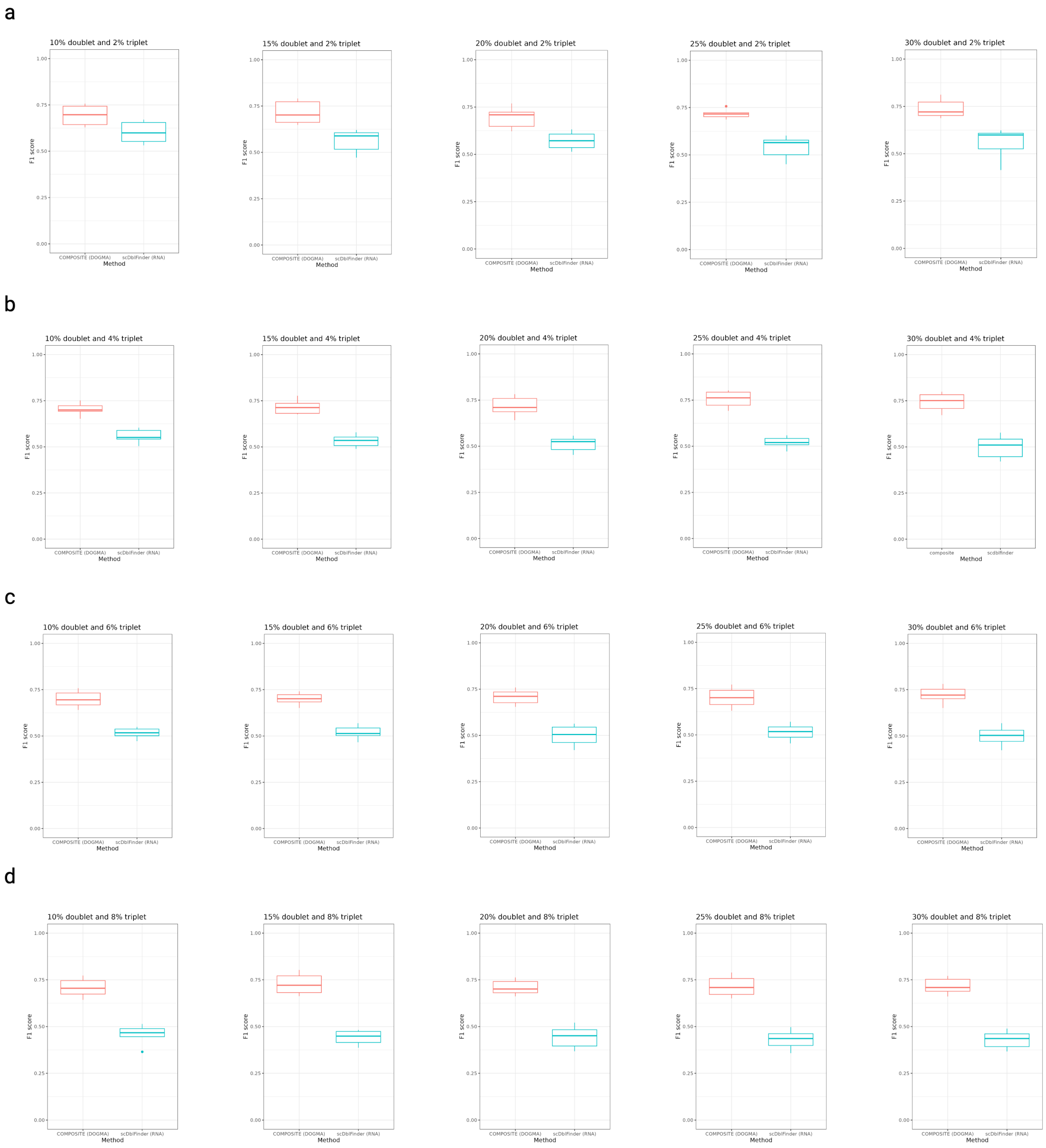

Figure S33: Benchmarking COMPOSITE (DOGMA) with scDblFinder (RNA) on simulated datasets with various doublet and triplet rates

a Doublet rates ranging from 10% to 30% and triplet rates fixed to 2%
b Doublet rates ranging from 10% to 30% and triplet rates fixed to 4%
c Doublet rates ranging from 10% to 30% and triplet rates fixed to 6%
d Doublet rates ranging from 10% to 30% and triplet rates fixed to 8%
The benchmarking metrics are F1 scores. In the boxplots, the box spans from the first to third quartile, with the median depicted as a line in the middle. The whiskers extend to 1.5 times the interquartile range (IQR).

a

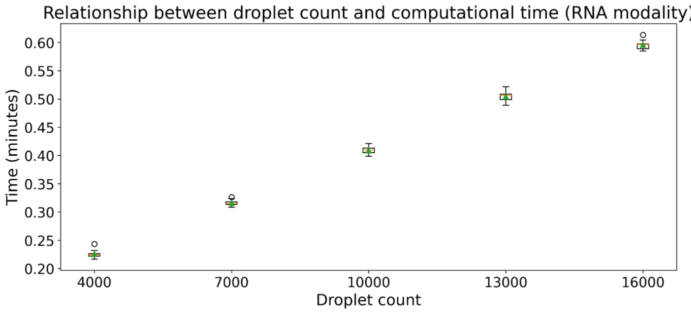

b

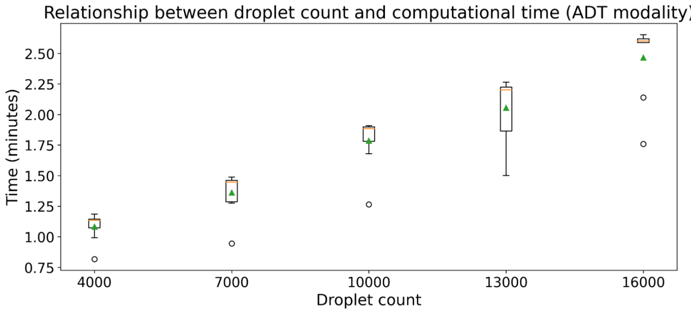

c

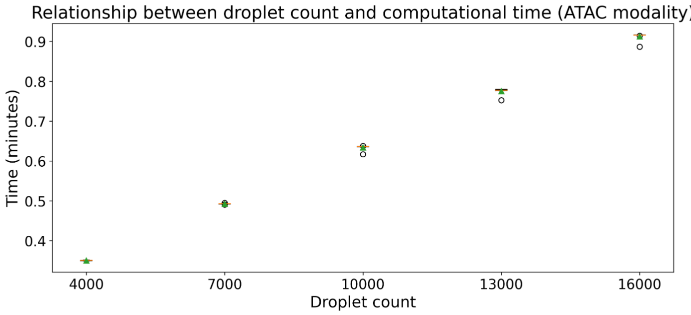

Figure S34: The relationships between the number of droplets in the dataset and the computational time of COMPOSITE

a-c The figure illustrates the relationships between the single modality computational time of COMPOSITE and the number of droplets in the dataset respectively for the RNA modality (**a**), the ADT modality (**b**), and the ATAC modality (**c**). Each boxplot represents the distribution of computational time for COMPOSITE using the corresponding number of randomly selected droplets from the 9 in-house datasets with over 16000 droplets. 300 stable features were used for the RNA and ATAC modalities, and 16 stable features were used for the ADT modality. In the boxplots, the box spans from the first to third quartile, with the median depicted as a line and the mean depicted as a triangle in the middle. The whiskers extend to 1.5 times the interquartile range (IQR). The GPU used for computing was an NVIDIA A100 PCIe.

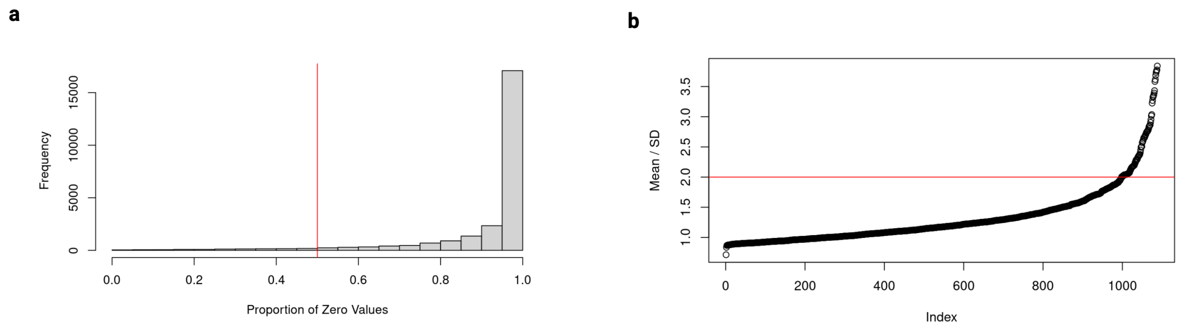

Figure S35: An illustration of stable feature selection criteria

a The first step of stable feature selection: select features with a low proportion of zero counts. In practice, we recommend using 50% as the threshold. **b** The second step: select features with a high mean/SD ratio.

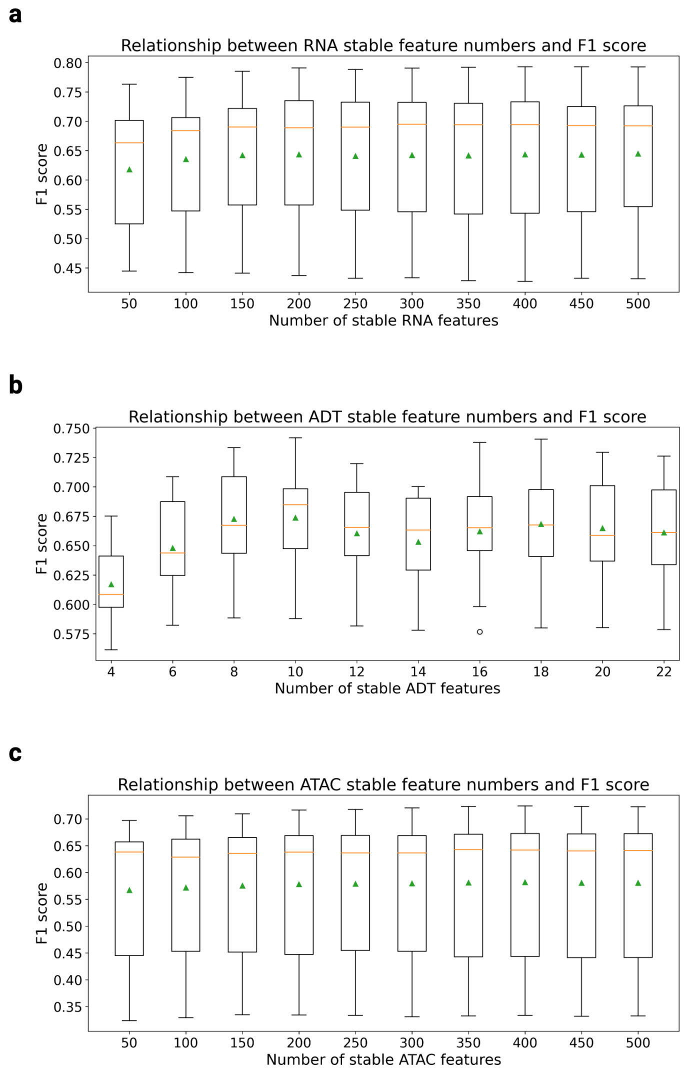

Figure S36: The relationships between the number of stable features and the single modality COMPOSITE performance

a-c The figure illustrates the relationships between the single modality prediction performance and the number of stable RNA features (**a**), stable ADT features (**b**), and stable ATAC features (**c**). Each boxplot represents the distribution of F1 scores achieved by the single modality prediction using the corresponding number of stable features on all 17 in-house datasets. In the boxplots, the box spans from the first to third quartile, with the median depicted as a line and the mean depicted as a triangle in the middle. The whiskers extend to 1.5 times the interquartile range (IQR).

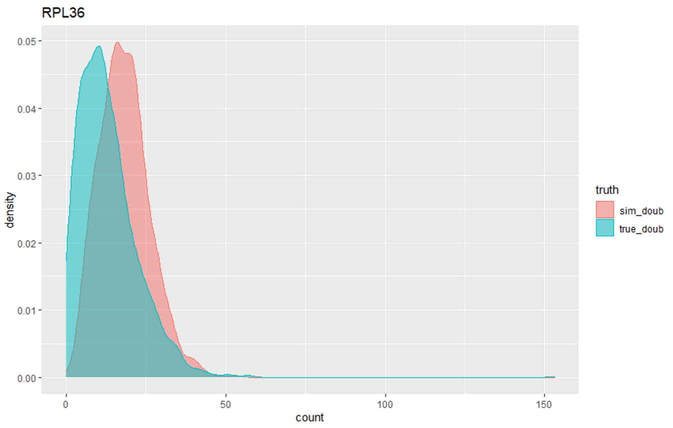

Figure S37: An illustration of the decline effect

The distribution of recorded *RPL36* (a stable RNA feature) expression levels in ground truth doublets versus that in simulated doublets, which were simulated by randomly summing expression profiles of two ground truth singlets. In general, the ground truth doublets had lower expression levels compared to the simulated doublets.

Supplementary Tables

Dataset	Experiment	Multiplet annotation	Sample type	ADT staining	Droplet number	Multiplet number	Multiplet rate
PB-1	DOGMA-seq	Cell hashing	Peripheral blood (T cell enriched)	TotalSeq-A_Human_Universal_Cocktail	13093	2783	0.213
PB-2					14512	2917	0.201
PB-3					14827	3375	0.228
PB-4					16979	3744	0.221
PB-5					20204	5823	0.288
PB-6					18625	4944	0.265
PB-7					20573	6056	0.294
PB-8					20604	7096	0.314
PB-9					19366	5752	0.297
PB-10					17353	3849	0.222
Ileum-1			Ileum (immune cell enriched)	TotalSeq-A_Human_Universal_Cocktail+EpCAM	19090	4657	0.244
Ileum-2					17412	3024	0.174
Ileum-3					13623	2701	0.198
Ileum-4					13969	2634	0.189
Ileum-5					12328	2202	0.179
Ileum-6					12321	1965	0.159
Ileum-7					15244	2842	0.186

Table S1: Benchmarking dataset summary

Sample	Modalities	F1 score (median[Q1, Q3])	AUPRC(median[Q1, Q3])
Peripheral blood (T cell enriched)	RNA	0.725[0.707, 0.750]	0.771[0.762, 0.793]
	ATAC	0.668[0.654, 0.696]	0.742[0.723, 0.762]
	ADT	0.684[0.674, 0.714]	0.743[0.727, 0.776]
	RNA+ATAC	0.771[0.735, 0.775]	0.767[0.750, 0.784]
	RNA+ADT	0.746[0.732, 0.772]	0.792[0.765, 0.812]
	DOGMA	0.754[0.741, 0.775]	0.794[0.780, 0.810]
Ileum (immune cell enriched)	RNA	0.511[0.467, 0.558]	0.591[0.550, 0.631]
	ATAC	0.438[0.376, 0.509]	0.487[0.424, 0.567]
	ADT	0.610[0.601, 0.647]	0.654[0.639, 0.699]
	RNA+ATAC	0.470[0.452, 0.548]	0.563[0.533, 0.626]
	RNA+ADT	0.621[0.616, 0.648]	0.688[0.658, 0.714]
	DOGMA	0.632[0.623, 0.660]	0.704[0.675, 0.733]

Table S2: Single-omics and multiomics prediction performance of COMPOSITE on the in-house DOGMA-seq datasets

Supplementary References

1. Hao, Y. et al. Integrated analysis of multimodal single-cell data. *Cell* **184**, 3573-3587 e3529 (2021).
