# Supplementary material for: A unified model-based framework for doublet or multiplet detection in single-cell multiomics data
Source: Nat Commun. 2024 Jul 2;15:5562. doi: 10.1038/s41467-024-49448-x (PMC11220103; doi:10.1038/s41467-024-49448-x)
Supplement: Supplementary file 3 — Reporting Summary [file 41467_2024_49448_MOESM3_ESM.pdf]

nature portfolio

Corresponding author(s): Wei Chen

Last updated by author(s): May 10, 2024

Reporting Summary

Nature Portfolio wishes to improve the reproducibility of the work that we publish. This form provides structure for consistency and transparency in reporting. For further information on Nature Portfolio policies, see our Editorial Policies and the Editorial Policy Checklist.

Statistics

For all statistical analyses, confirm that the following items are present in the figure legend, table legend, main text, or Methods section.

n/a	Confirmed	
☐	☒	The exact sample size (*n*) for each experimental group/condition, given as a discrete number and unit of measurement
☐	☒	A statement on whether measurements were taken from distinct samples or whether the same sample was measured repeatedly
☒	☐	The statistical test(s) used AND whether they are one- or two-sided<br>*Only common tests should be described solely by name; describe more complex techniques in the Methods section.*
☒	☐	A description of all covariates tested
☐	☒	A description of any assumptions or corrections, such as tests of normality and adjustment for multiple comparisons
☐	☒	A full description of the statistical parameters including central tendency (e.g. means) or other basic estimates (e.g. regression coefficient) AND variation (e.g. standard deviation) or associated estimates of uncertainty (e.g. confidence intervals)
☒	☐	For null hypothesis testing, the test statistic (e.g. *F*, *t*, *r*) with confidence intervals, effect sizes, degrees of freedom and *P* value noted<br>*Give P values as exact values whenever suitable.*
☒	☐	For Bayesian analysis, information on the choice of priors and Markov chain Monte Carlo settings
☒	☐	For hierarchical and complex designs, identification of the appropriate level for tests and full reporting of outcomes
☒	☐	Estimates of effect sizes (e.g. Cohen's *d*, Pearson's *r*), indicating how they were calculated

Our web collection on statistics for biologists contains articles on many of the points above.

Software and code

Policy information about availability of computer code

Data collection	All DOGMA-seq, bulk RNA-seq, and bulk ATAC-seq libraries were generated in our laboratory, and the sequencing was done on the Illumina NovaSeq 6000 System (DOGMA-seq libraries), Illumina NextSeq 500 System (bulk RNA-seq libraries and some bulk ATAC-seq libraries), or Illumina HiSeq 2500 System (some bulk ATAC-seq libraries). The raw sequencing data were downloaded to a file storage array attached to the University of Pittsburgh's high throughput computing cluster or to our server.<br>Preprocessing ATAC FASTQ files to get UMI counts matrices: Cell Ranger Arc v2.0.2<br>Preprocessing RNA and ADT FASTQ files to get UMI counts matrices: KITE v0.46<br>R packages:Seurat (version 4.1.0), Signac v1.12.0
Data analysis	The data analysis tool proposed in this paper, COMPOSITE, has been implemented in the “sccomposite” Python package, which is available at https://github.com/CHPGenetics/COMPOSITE. The COMPOSITE tool is also available as a cloud-based application https://shiny.crc.pitt.edu/shinyproj_composite/.

For manuscripts utilizing custom algorithms or software that are central to the research but not yet described in published literature, software must be made available to editors and reviewers. We strongly encourage code deposition in a community repository (e.g. GitHub). See the Nature Portfolio guidelines for submitting code & software for further information.

Data

Policy information about availability of data

All manuscripts must include a data availability statement. This statement should provide the following information, where applicable:

- Accession codes, unique identifiers, or web links for publicly available datasets
- A description of any restrictions on data availability
- For clinical datasets or third party data, please ensure that the statement adheres to our policy

The cell hashing data and the single-cell stable feature data generated in this study have been deposited in Zenodo under accession code DOI 10.5281/zenodo.11167173 [https://doi.org/10.5281/zenodo.11167174]. Source data are provided with this paper.

Research involving human participants, their data, or biological material

Policy information about studies with human participants or human data. See also policy information about sex, gender (identity/presentation), and sexual orientation and race, ethnicity and racism.

Reporting on sex and gender	Both male and female study subjects were included in our study. Sex was self-reported. We did not perform sex- or gender-based analyses.
Reporting on race, ethnicity, or other socially relevant groupings	Our study was limited to study subjects with self-reported white, European ancestry because the vast majority of currently known genetic risk loci in inflammatory bowel disease and other chronic immune-mediated inflammatory diseases were discovered in white, European ancestry populations.
Population characteristics	Study subject inclusion criteria included a) self-reported white, European ancestry (because the vast majority of currently known genetic risk loci in inflammatory bowel disease and other chronic immune-mediated inflammatory diseases were discovered in white, European ancestry populations), b) ages 18-35 (to limit confounding from effects of aging on the immune system, and so that the age range of study subjects would be comparable to the peak age of IBD onset in adults), and c) no history of chronic, immune-mediated disease or use of immunomodulatory/biologic medications to treat a chronic condition (to avoid confounding from effects of immunomodulator and biologic therapies used to treat patients with immune-mediated diseases on the T cell responses that we aimed to measure).
Recruitment	The University of Pittsburgh Pitt+Me research advertising and referral service that matches individuals to research studies based on search criteria was used to identify potential study subjects who were then contacted by clinical research coordinators to verify eligibility and recruit eligible study subjects.
Ethics oversight	All research activities were reviewed and approved by the University of Pittsburgh Institutional Review Board.

Note that full information on the approval of the study protocol must also be provided in the manuscript.

Field-specific reporting

Please select the one below that is the best fit for your research. If you are not sure, read the appropriate sections before making your selection.

☒ Life sciences ☐ Behavioural & social sciences ☐ Ecological, evolutionary & environmental sciences

For a reference copy of the document with all sections, see nature.com/documents/nr-reporting-summary-flat.pdf

Life sciences study design

All studies must disclose on these points even when the disclosure is negative.

Sample size	No statistical methods were used to predetermine sample size. Large numbers of cells in each individual constitute a sufficient sample size for accurate estimation.
Data exclusions	No data were excluded from the analyses.
Replication	In this study, we applied our method on 19 real datasets. Our method can always provide stable results in each replication on all datasets. The relevant results and the code to reproduce the results are publicly available at https://github.com/CHPGenetics/COMPOSITE/tree/main/experiments.
Randomization	Randomization is not relevant to our study. Methods involved in this study focus on analyzing data from only one experimental group/condition.
Blinding	Blinding is not relevant to our study. Methods involved in this study focus on analyzing data from one experimental group/condition.

Reporting for specific materials, systems and methods

We require information from authors about some types of materials, experimental systems and methods used in many studies. Here, indicate whether each material, system or method listed is relevant to your study. If you are not sure if a list item applies to your research, read the appropriate section before selecting a response.

Materials & experimental systems

n/a	Involved in the study
[ ]	[x] Antibodies
[x]	[ ] Eukaryotic cell lines
[x]	[ ] Palaeontology and archaeology
[x]	[ ] Animals and other organisms
[x]	[ ] Clinical data
[x]	[ ] Dual use research of concern
[x]	[ ] Plants

Methods

n/a	Involved in the study
[x]	[ ] ChIP-seq
[x]	[ ] Flow cytometry
[x]	[ ] MRI-based neuroimaging

Antibodies

Antibodies used

1. TotalSeq™-A Human Universal Cocktail, V1.0, catalog number 399907 (BioLegend)
2. TotalSeq™-A0251 anti-human Hashtag 1 Antibody, catalog number 394601 (BioLegend)
3. TotalSeq™-A0252 anti-human Hashtag 2 Antibody, catalog number 394603 (BioLegend)
4. TotalSeq™-A0253 anti-human Hashtag 3 Antibody, catalog number 394605 (BioLegend)
5. TotalSeq™-A0254 anti-human Hashtag 4 Antibody, catalog number 394607 (BioLegend)
6. TotalSeq™-A0255 anti-human Hashtag 5 Antibody, catalog number 394609 (BioLegend)
7. TotalSeq™-A0256 anti-human Hashtag 6 Antibody, catalog number 394611 (BioLegend)
8. TotalSeq™-A0257 anti-human Hashtag 7 Antibody, catalog number 394613 (BioLegend)
9. TotalSeq™-A0258 anti-human Hashtag 8 Antibody, catalog number 394615 (BioLegend)
10. TotalSeq™-A0259 anti-human Hashtag 9 Antibody, catalog number 394617 (BioLegend)
11. TotalSeq™-A0260 anti-human Hashtag 10 Antibody, catalog number 394619 (BioLegend)
12. TotalSeq™-A0262 anti-human Hashtag 12 Antibody, catalog number 394623 (BioLegend)
13. TotalSeq™-A0263 anti-human Hashtag 13 Antibody, catalog number 394625 (BioLegend)
14. TotalSeq™-A0264 anti-human Hashtag 14 Antibody, catalog number 394627 (BioLegend)
15. TotalSeq™-A0265 anti-human Hashtag 15 Antibody, catalog number 394629 (BioLegend)
16. TotalSeq™-A0276 anti-human Hashtag 16 Antibody, catalog number 394681 (BioLegend)
17. PerCP mouse anti-human CD4, catalog number 300528 (BioLegend)
18. APC mouse anti-human CD45RO, catalog number 559865 (BD Pharmingen)
19. BV421 mouse anti-human CD196, catalog number 562515 (BD Pharmingen)

Validation

1. https://www.biolegend.com/en-us/products/totalseq-a-human-universal-cocktail-v1-20321
2. https://www.biolegend.com/en-us/products/totalseq-a0251-anti-human-hashtag-1-16080
3. https://www.biolegend.com/en-us/products/totalseq-a0252-anti-human-hashtag-2-antibody-16081
4. https://www.biolegend.com/en-us/products/totalseq-a0253-anti-human-hashtag-3-antibody-16084
5. https://www.biolegend.com/en-us/products/totalseq-a0254-anti-human-hashtag-4-antibody-16086
6. https://www.biolegend.com/en-us/products/totalseq-a0255-anti-human-hashtag-5-antibody-16088
7. https://www.biolegend.com/en-us/products/totalseq-a0256-anti-human-hashtag-6-antibody-16089
8. https://www.biolegend.com/en-us/products/totalseq-a0257-anti-human-hashtag-7-antibody-16090
9. https://www.biolegend.com/en-us/products/totalseq-a0258-anti-human-hashtag-8-antibody-16092
10. https://www.biolegend.com/en-us/products/totalseq-a0259-anti-human-hashtag-9-antibody-16093
11. https://www.biolegend.com/en-us/products/totalseq-a0260-anti-human-hashtag-10-antibody-16094
12. https://www.biolegend.com/en-us/products/totalseq-a0262-anti-human-hashtag-12-antibody-16095
13. https://www.biolegend.com/en-us/products/totalseq-a0263-anti-human-hashtag-13-antibody-16096
14. https://www.biolegend.com/en-us/products/totalseq-a0264-anti-human-hashtag-14-antibody-16097
15. https://www.biolegend.com/en-us/products/totalseq-a0265-anti-human-hashtag-15-antibody-16098
16. https://www.biolegend.com/en-us/products/totalseq-a0276-anti-human-hashtag-16-antibody-21620
17. https://www.biolegend.com/en-us/products/percp-anti-human-cd4-antibody-4215
18. https://www.bdbiosciences.com/content/bdb/paths/generate-tds-document.us.559865.pdf
19. https://www.bdbiosciences.com/content/bdb/paths/generate-tds-document.us.562515.pdf

Plants

Seed stocks	*Report on the source of all seed stocks or other plant material used. If applicable, state the seed stock centre and catalogue number. If plant specimens were collected from the field, describe the collection location, date and sampling procedures.*
Novel plant genotypes	*Describe the methods by which all novel plant genotypes were produced. This includes those generated by transgenic approaches, gene editing, chemical/radiation-based mutagenesis and hybridization. For transgenic lines, describe the transformation method, the number of independent lines analyzed and the generation upon which experiments were performed. For gene-edited lines, describe the editor used, the endogenous sequence targeted for editing, the targeting guide RNA sequence (if applicable) and how the editor was applied.*
Authentication	*Describe any authentication procedures for each seed stock used or novel genotype generated. Describe any experiments used to assess the effect of a mutation and, where applicable, how potential secondary effects (e.g. second site T-DNA insertions, mosiacism, off-target gene editing) were examined.*
